# NetConfer: a web application for comparative analysis of multiple biological networks

**DOI:** 10.1186/s12915-020-00781-9

**Published:** 2020-05-19

**Authors:** Sunil Nagpal, Krishanu Das Baksi, Bhusan K. Kuntal, Sharmila S. Mande

**Affiliations:** 1 Bio-Sciences R&D Division, TCS Research, Tata Consultancy Services Ltd., 54-B Hadapsar Industrial Estate, Pune, 411 013 India; 2 Chemical Engineering and Process Development Division, CSIR-National Chemical Laboratory, Dr. Homi Bhabha Road, Pune, 411 008 India; 3 Academy of Scientific and Innovative Research (AcSIR), CSIR-National Chemical Laboratory Campus, Pune, 411 008 India

**Keywords:** Biological networks, Network comparison, Interaction networks, Visualization, Bioinformatics

## Abstract

**Background:**

Most biological experiments are inherently designed to compare changes or transitions of state between conditions of interest. The advancements in data intensive research have in particular elevated the need for resources and tools enabling comparative analysis of biological data. The complexity of biological systems and the interactions of their various components, such as genes, proteins, taxa, and metabolites, have been inferred, represented, and visualized via graph theory-based networks. Comparisons of multiple networks can help in identifying variations across different biological systems, thereby providing additional insights. However, while a number of online and stand-alone tools exist for generating, analyzing, and visualizing individual biological networks, the utility to batch process and comprehensively compare multiple networks is limited.

**Results:**

Here, we present a graphical user interface (GUI)-based web application which implements multiple network comparison methodologies and presents them in the form of organized analysis workflows. Dedicated comparative visualization modules are provided to the end-users for obtaining easy to comprehend, insightful, and meaningful comparisons of various biological networks. We demonstrate the utility and power of our tool using publicly available microbial and gene expression data.

**Conclusion:**

NetConfer tool is developed keeping in mind the requirements of researchers working in the field of biological data analysis with limited programming expertise. It is also expected to be useful for advanced users from biological as well as other domains (working with association networks), benefiting from provided ready-made workflows, as they allow to focus directly on the results without worrying about the implementation. While the web version allows using this application without installation and dependency requirements, a stand-alone version has also been supplemented to accommodate the offline requirement of processing large networks.

## Background

Networks provide one of the means to construct a theoretical model for representing the relationships between the entities of complex systems. Such representations often help visualizing in a convenient way the relationships as well as capture the concepts for an improved understanding. Networks can also be used to transform a generic problem into a mathematical model represented by graphs. Consequently, methods and tools used for solving a graph theory problem can easily be applied to augment the already available set of solutions. For biological systems, the use of network-based methods finds a wide range of applications for systems level understanding [1–4]. A biological network can be generated using known or predicted associations between various components of the system (like genes, proteins, microbial taxa, and metabolites). With the availability of large-scale multi-omics data, the current research focus in biological systems has widened from understanding individual level variations to comparison of variations across systems (like different ecological environments, multiple organisms, and multiple stages of a developmental cycle). Multiple network comparisons provide a means to gather improved insights into the variations across a number of such systems which are each represented by a network.

Cytoscape [5], backed by a multitude of plugins across diverse categories, provides the most feature-rich interface for biological network analysis. Although “network comparison” is an active category of Cytoscape plugins, most of them provide niche analytical workflows often useful for a specific category of networks [6, 7]. Another stand-alone tool, called CompNet [8], also provides methodologies applicable for analyzing multiple biological interaction networks. With rapid advancements in web-based technologies and significant improvement in network bandwidth, modern web-based tools provide a convenient alternative to stand-alone software with advantages like platform independence, no installation/updating requirements, and access from anywhere. Although a number of web-based tools, like tYNA [9], NeAT [10], PINA [6], OmicsNet [11], BINA [12], NAP [13], CellMaps [14], GraphSpace [15], NetworkAnalyst [16], and NetVenn [17], have been developed for analyzing specialized metabolic or interactome networks, they have certain limitations pertaining to comparison of multiple networks. For example, Network Analysis Tool (NeAT) mainly focuses on network clustering and is limited to comparison of two networks based on intersection and union of network components. On the other hand, Network Analysis Provider (NAP) allows comparison of mainly local graph properties across multiple networks. A detailed comparison of the above tools with NetConfer is provided in Additional file 1: Table 1.

**Fig. 1:**
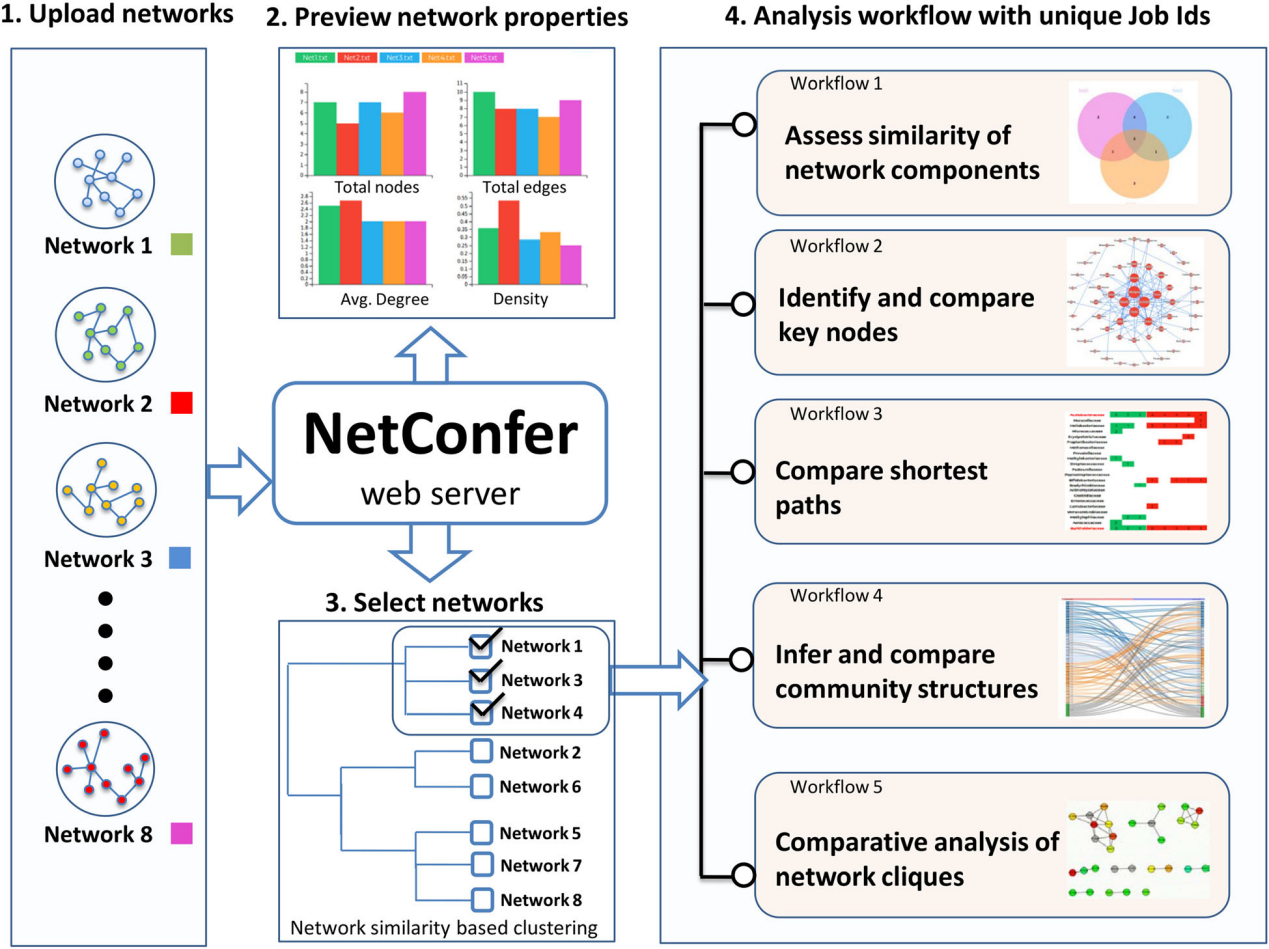
Description of the overall methodology for multiple network comparison using NetConfer starting from network selection, preview, and application of workflows to a selection

In this communication, we present a web-based tool (called NetConfer) that implements various network comparison methodologies and present them in the form of organized analysis workflows for comparing various types of biological networks (Fig. 1). Each workflow is designed to achieve a specific network analysis objective. These include comparison of network components, identification of union/intersection/exclusive nodes or edges, shortest path comparison, community and clique analysis, and comprehensive network visualization modules. Events like network rewiring in different stages of progression of disease, topological property changes in time series networks, and comparing networks belonging to different populations are some examples where NetConfer can be extremely useful. We expect our tool to be a valuable contribution in the field of network and systems biology.

## Methods

NetConfer represents an ensemble of workflows designed to provide a structured approach for exploring multiple (biological) networks as well as comparison through several classical and innovative visualization techniques. While the backend algorithms are coded using Python with JavaScript and PHP for web components, D3.js (https://d3js.org/) and Cytoscape.js [18] have been used for the frontend modules. Networkx python library (https://networkx.github.io) and SNAP C++ library [19] components have been employed in the backend for reliable and standard computations of individual network parameters/properties. Given the “multi-network” processing objective of the platform, end-users are allowed to supply multiple network files (maximum number of 8 networks) as delimited edge lists (Additional file 2: Fig. S1). Various options like specifying the columns corresponding to the source and target, delimiter selection, and edge weight can be easily specified from the input form. It is pertinent to note that all the input network files in a given submission should be derived from a similar type of data like multiple networks of genes, proteins, and microbes. In addition, the compared networks are expected to share at least some nodes in common to obtain meaningful insights. Nevertheless, NetConfer results are not biased by the nature of data and the above recommendations are solely meant for optimal testing of various features of the tool.

Upon successful upload of networks, an interactive grouped bar chart-based preview of four global network properties (i.e., total nodes, total edges, cluster coefficient, and density) of all the submitted networks is generated, thereby enabling users to gather an initial idea of the input network structures (Additional file 2: Fig. S2). A status terminal in the user interface displays errors encountered while performing the background tasks. Users can modify labels and colors associated with each network by assessing the color and label maps in the preview (Additional file 2: Fig. S3). Upon proceeding, the networks are automatically clustered based on their overall similarity (using edge Jaccard index) and presented to users in the form of an interactive tree/dendogram for ease of selection and subsequent application of workflows and visualizations (Additional file 2: Fig. S4). The working area is provided to the end-users in the form of a personalized dashboard comprising of “Workflows Dashboard” and “Visualization Dashboard.” To start any analysis in Workflows or Visualizations Dashboard, users get the option of selecting two or more networks from the clustered tree. In addition to the checkbox-based selection feature on the tree, clicking on nodes of interest from the tree redirects users to the graphical representation of the given network, wherein users get to interact, analyze, and customize the node-specific network graph.

### Job management system

NetConfer provides an efficient job management system comprising of following components:

- Unique identifier-based task initiation and recording
- Tagging of unique identifier to a personalized dashboard specific to the job and end-users
- User specific local job history management
- Search and access
- No requirement for registration or sharing of personal information

Each submission on NetConfer application web server is assigned to a 10-character unique alpha-numeric code (termed as JOB ID) and is displayed to the user for record keeping. The same is also locally stored in the user (and browser)-specific “Job History” page of NetConfer, wherein the user can keep track of his/her personal submissions. Post-submission of data and generation of JOB ID, the user is redirected to a personal “Workflows” dashboard. This dashboard along with all associated modules can be accessed by using the provided unique JOB ID. The search and access can be accomplished through the dedicated Job History page or through the Job Search widget provided on “Home Page” and “Submissions” section of NetConfer. It is pertinent to note that NetConfer purges a job and its associated dashboard(s) after 7 days of completion of the job. This unique “Job Management System,” coupled with personalized dashboards, ensures that users are able to use the platform for tracking their tasks and re-assess their workflows without needing them to register or share of any personal information. In addition, the provision for live status terminal in NetConfer allows an end-user to get dynamic updates about status of the task being performed. This is coupled by providing information about the time taken for each task and is expected to enhance the user experience.

### Workflows and modules

The available methodologies and visualizations in NetConfer can be broadly classified into two categories, each tagged to a dedicated personalized dashboard:

(A) Analysis workflows
(B) Visualization modules

#### Analysis workflows

This category of analyses provides five mutually exclusive, yet logically connected multiple network analysis workflows and is described below (Additional file 2: Fig. S5).

##### Assessing similarity of network components

Identification of common nodes and edges among a set of networks is one of the primary features considered while comparing multiple networks. Therefore, the first workflow has been designed to identify nodes as well as edges which are commonly shared between two or more of the selected set of networks. The results can be viewed using the following three ways:

(i) The first method provides Venn diagrams augmented with interactive user operations to graphically display similarities and differences across user-chosen networks [20]. The overlapping regions correspond to the intersection of multiple networks, with the number depicting the number of common nodes/ edges among the intersecting networks. The list of common edges/nodes among two or more networks can be easily identified by clicking on the numbers displayed on the corresponding intersecting regions (Additional file 2: Fig. S6, S7).
(ii) The second method uses a powerful visualization technique called “upset plot” which displays the number of common nodes and edges across all combinations of networks [21]. The filled and connected circles in the lower part of the upset plot correspond to different combinations of the networks which are being considered, and the bar heights indicate the number of common edges of the corresponding combinations (Additional file 2: Fig. S8, S9). Clicking on any bar provides the corresponding common nodes/edges of the selected combination set in form of a list or circular graph. If an “upset plot” of nodes is selected, only the constituent nodes for the combination are highlighted in the circle graph. Selecting a combination bar from the “upset plot” of edges shows the resultant network as a circle graph of the constituent nodes and edges. The generated “upset plots” can be sorted either by the size of the combinations (combination cardinality) or by the sets (set cardinality) (Additional file 2: Fig. S10).
(iii) The third method uses the classical, hitherto highly interactive, customizable, and downloadable network diagrams to visualize exclusive and intersecting set of edges between selected set of networks. This functionality can be accessed through the visualization modules presented in Visualization Dashboard discussed in a later section.

*Biological use*. This workflow can enable clinicians/researchers to easily obtain an overall idea of the similarities/dissimilarities between the uploaded networks. If two or more networks are very similar with respect to the constituent nodes, the values in the intersecting regions of the Venn diagram will be helpful to quantify as well as view the same. For example, the common genes perturbed in the virulent and avirulent infections could be identified using the Venn diagram of nodes in case study 2 (described later in the manuscript). Events like network rewiring can also be inferred for an uploaded set of networks if their constituent nodes appear very similar but show significant differences in the Venn diagram of edges.

##### Identifying and comparing key nodes

One of the most common ways of comparing networks is by studying different properties (or centrality measures, also termed as local properties) of the nodes and global properties of the network (Additional file 2: Fig. S11). NetConfer provides a dedicated workflow for assessing and comparing various global and local properties of all selected networks. Some of the most useful (local) properties of nodes in a network covered by NetConfer are degree, betweenness centrality, hub and authority score, eccentricity, and eigenvector centrality [4]. The workflow allows tabulated and graphical analysis and tracing of various properties across selected set of networks. These properties can be viewed by sortable and searchable tables for comparing the node properties across different networks. Further, the table can be generated by either having the values of one of the properties of a node across different networks or having the different properties of all the nodes in one network. The first option is useful for understanding the changes in centrality measure across different networks. Additionally, NetConfer utilizes interactive parallel coordinates called “Delta Centrality” for providing an innovative way of viewing changes in various centrality measures (Additional file 2: Fig. S12). A user can choose a centrality measure of interest by using the radio button and highlight the values for one or more selected node using the tabulated summary by clicking on the desired node name. This feature is useful especially for comparing spatio-temporally ordered networks.

*Biological use*. The centrality measures of various nodes in a network can be used for identifying critical or key nodes in a network. For example, in a microbial association network, degree and betweenness might be useful to identify key nodes (“microbes”) that help in microbial communication. Nodes with high betweenness score are essentially key points of information flow and if removed can disrupt the whole network. Similarly, hub and authority scores have been proven to be useful in identification of essential proteins in protein-protein interaction networks.

##### Comparing shortest paths

NetConfer allows a user to perform a comparative analysis of shortest paths between a given pair of “source” and “target” node across the selected set of networks using a novel interactive layout (Additional file 2: Fig. S13). The layout not only allows comparing multiple shortest paths within a network, but also across a selected set of networks in an easy and intuitive way. In the figure, the colors correspond to different networks (there may be more than one shortest path between two nodes in a single network), and the numbers correspond to the order of the nodes in the shortest path. The source and target nodes (as chosen by the user in the workflow) are always positioned at the bottom and top of the graph, respectively. Using this visualization, NetConfer makes it easy to identify nodes which are consistently present along the shortest path (indicating the preferred nodes) between the “source” and “target” nodes across different networks.

*Biological use*. Identification of shortest paths is useful during analyses of a range of biological networks like metabolic pathway analysis [22], alterations in protein-protein interaction networks [23], and order of interaction cascades in transcription factor networks [24]. Nodes which are consistently present in the shortest path between the “source” and “target” across different networks are likely to play an important role in the dynamics of the system. For example, in case study 2, gene “NCAPG” forms an important connecting member for the multiple differential shortest paths identified between genes “BIRC5” and “ASPM” (details are described later in the manuscript).

##### Inferring and comparing community structures

This workflow can be used to find and compare communities in a selected set of networks using innovative plots and tables. NetConfer offers a novel way of tracking changes in community structures across a pair of networks. Additional file 2: Fig. S14 represents an example of a heatmap-embedded Sankey diagram-based community transition tracking utility of this workflow. In both the vertical axes, the communities (which are easily distinguishable by colors) along with their constituent member nodes are ordered in the descending order of their size. Using the “node to node” flow between the two vertical axes, changes in communities’ constituent can be tracked easily, thereby helping users in identifying not only communities which are conserved across networks, but also the ones which undergo reshuffling. Heatmap embeddings besides the nodes represent the three important centrality measures, i.e., degree, hub score, and betweenness (whose values have been rank normalized across the given pair of network). This feature allows easy identification of key nodes and tracking their fate in communities of the two networks being compared. Additionally, a tabulated summary of the “community shuffling” (with an intersection and Jaccard score of community similarity) is also presented for user convenience. The results are also depicted in a “comprehensively searchable” tabulated layout for enabling users in identifying the communities that comprise of nodes (or group of nodes) of their interest (Additional file 2: Fig. S15). Highlighting of the searched query further makes the results easy to comprehend. By default, the tabulated searchable and sortable layout enlists communities in the descending order of their size additionally coloring them based on the parent network. By clicking on the community in the table, a network visualization (described in detail under the “Visualization modules” section) tab pops up displaying the community as a subnetwork (Additional file 2: Fig. S15).

*Biological use*. Closely linked hubs of interacting nodes represent a network community. Such communities provide important insights into the functional components and organization pattern of a biological system. For example, in a microbial association network, these modular hubs may constitute groups of microbes interdependent on each other for various functions. Understanding the nature and change in communities can hence be of great biological significance for community engineering experiments, understanding functional potential, pathogen colonization, etc. Further, understanding the changes in community structure across various states of a system (represented as a network) might help in identification of crucial “drivers” of the change [1].

##### Analysis and comparison of network cliques

This workflow is designed to identify and compare “cliques” between a selected set of input networks (Additional file 2: Fig. S16). Results are provided in “searchable, sortable, highlight-enabled” tabulated framework, similar to the ones implemented in community workflow. Users can choose nodes of interest (or a combination thereof) to explore cliques across all the chosen networks for comparison. In order to aid visual analyses of the results, users have the option to view the individual cliques within the networks by simply clicking on the clique names. Like the previous workflow, users can track the members of the cliques in other networks as well, which is facilitated by coloring the member nodes as gray and keeping the non-member nodes with the initial color (at the start of analysis). Another additional feature of the visualization is the ability to click and drag the clique member nodes together, which eases the ability to view the member nodes and their connections (describes later as a part of the case study).

*Biological use*. Clique (closely knit subset of nodes in a graph such as dyads, triads, and tetrads) serve as useful indicators for identifying co-expressed genes, finding (and comparing) motifs, protein complexes, and functional modules from protein-protein interaction (PPI) networks and for understanding microbial symbiosis. These small subunits are similar to communities but are often more robust indicators of biological subunits in a system [25].

#### Visualization modules

Apart from the workflows for network comparison as described above, NetConfer allows users to visually compare the results in a variety of ways, as described below:

In the “Visualization Dashboard” (Additional file 2: Fig. S17), individual network can be viewed by clicking on the network names in the hierarchically clustered tree. The visualization offers users options to customize and interact with the network visualization using simple and intuitive operations like dynamic change of node, font and edge size, and network layouts. In addition, end-users can also overlay network properties like degree, betweenness, and coreness to proportionally size the nodes of the network. Along with the above module for viewing the networks individually, NetConfer also provides modules wherein users have the option to choose two or more networks and view subsets or supersets of the networks. All these modules provide customizable and interactive subsets/supersets. The modules and their utilities are described below:

1. *Intersection visualization module*. In this module, the edges which are common across all selected networks can be viewed. All the features applicable to the network view, as described above, can be applied to analyze the intersection network.
2. *Exclusive visualization module*. In this module, the edges present exclusively in each of the selected networks as compared to all the other selected networks can be visualized. Nodes are colored according to their presence in different selected networks and can be customized for size, font, and layout as well.
3. *Union visualization module*. Using this module, the nodes and edges present across all the selected networks can be visualized. To help users understand which network the nodes belong to, every node is colored like a pie chart, with the colors corresponding to the networks in which the node is present. In addition, the edges are also given multiple colors to identify the networks they are found in. For example, the node A in Additional file 2: Fig. S18 has 5 colors indicating its presence across all five networks, whereas node L has only one color, implying its presence only in one network (net 5). On the other hand, the edge between node D and node S is present in two networks as determined by the colors (net 3 and net 5). Similarly, since the edge between node S and node A has only one color, this node is present in one network (net 1).
4. *Property mapped individual network visualization module*. Visualization of the nodes along with their local properties like degree, betweenness, and closeness are often of interest to users. In order to facilitate this, NetConfer offers a network visualization wherein all the different properties of the nodes can be used to size the nodes. For example, in Additional file 2: Fig. S19, the different nodes of network 1 (selected from the dropdown) are sized based on their degrees. Hence, it would be easier to identify the important nodes as well as their connections. The layout can be modified using the network layout (random, grid, concentric, hierarchical, and degree sorted circular layout) and property modifier, and the view can be zoomed using the zoom-in and zoom-out buttons in the network view modifier.
5. *Distance from the global union network*. A simple yet useful utility to visualize the distance of the individual network from the global union is implemented in this module. The distances are calculated as Jaccard indices [26] of the nodes and edges of the respective networks from the global union and presented as a radar chart. The network names are displayed as dimensional anchors placed equidistant on the periphery of a circle. The points on the radial line connecting the center of the circle to each network represent the corresponding distance (node and edge displayed in orange and green color respectively) of that network from the global union (Additional file 2: Fig. S20).

**Fig. 2:**
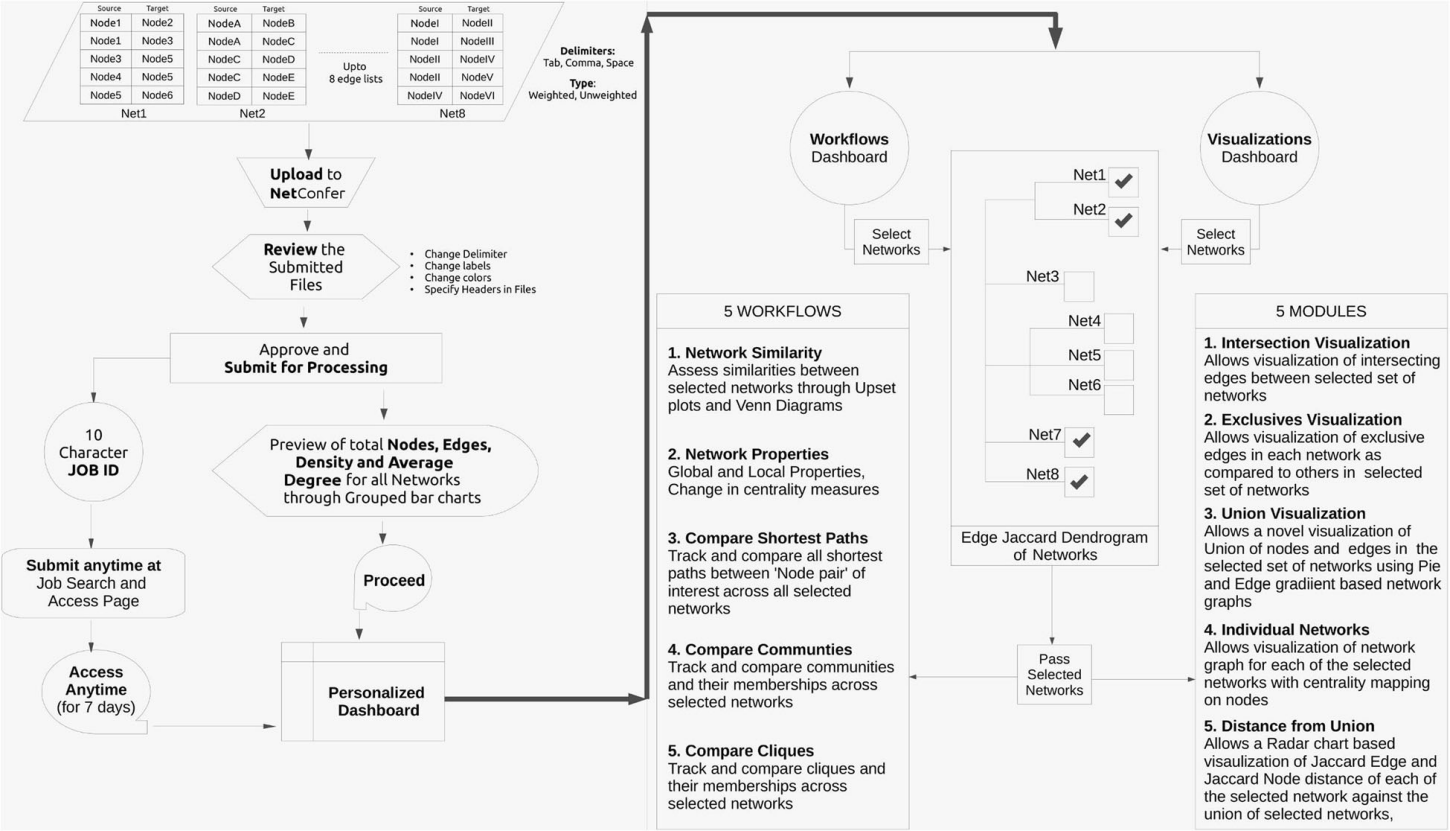
A flowchart summary of all the steps associated with requirements and submission of a task/job to NetConfer

**Fig. 3:**
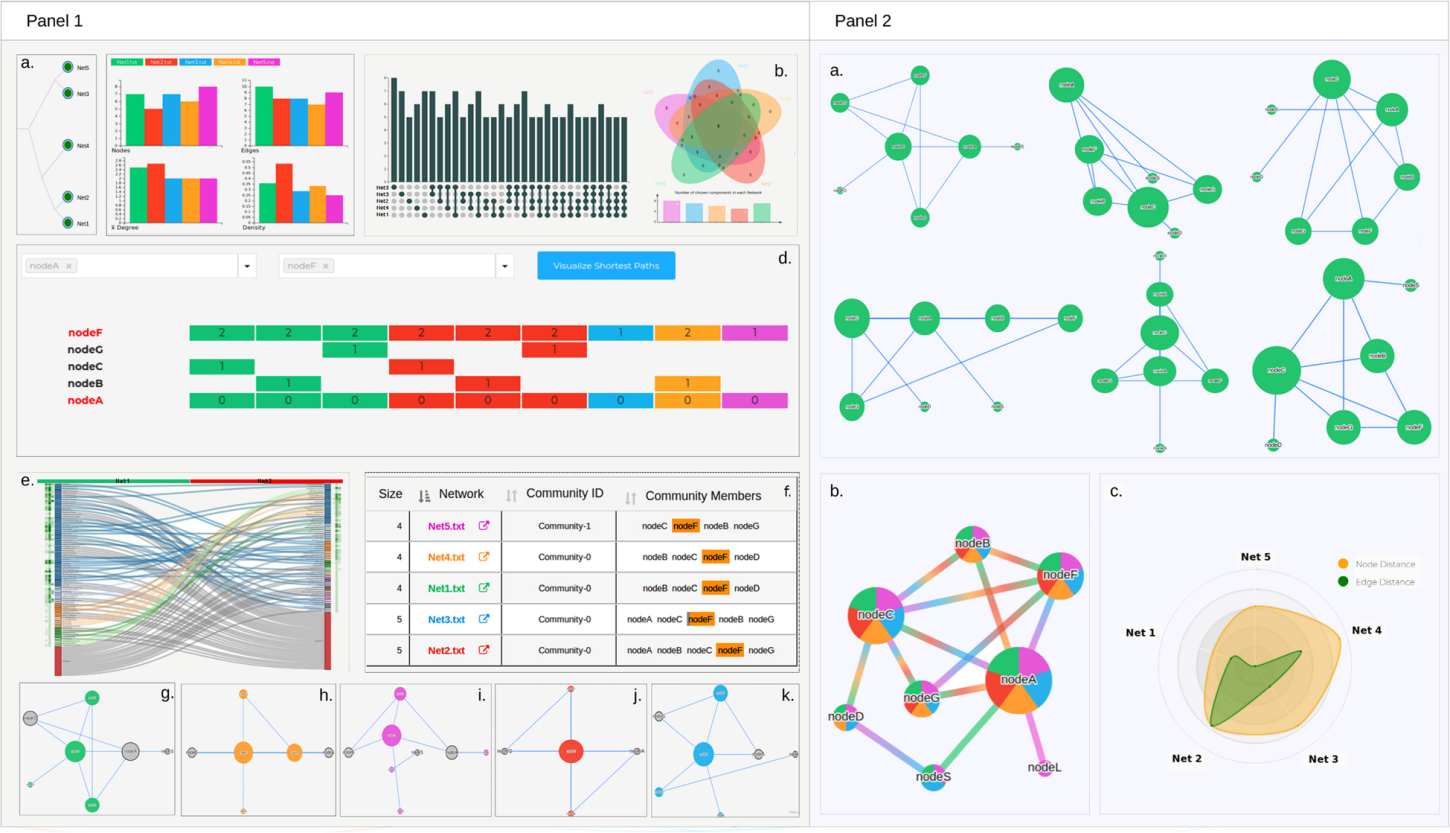
A graphical summary of various key visualizations and associated analyses possible using NetConfer. Panel 1 shows graphs pertaining to the Workflows Dashboard, while panel 2 depicts the graphical results offered by the Visualization Dashboard of NetConfer. In panel 1, figure (a) is a typical layout of the Edge-Jaccard index-based network dendogram containing selectable (checkbox) nodes; (b) represents the grouped bar chart-based “global property preview” generated, immediately after submission of various network files to NetConfer; (c) highlights the upset plot offered in NetConfer for set-similarity analysis (classical Venn diagrams are also offered as alternative); (d) represents the novel yet simple visualization approach for tracing shortest paths for a given pair of source and target nodes across networks of interest; (e) depicts the novel Sankey-heatmap coupled community transition visualization designed for tracing the changes in community memberships and centrality measure of the members between a given pair of networks; and (f) represents the tabulated visualization of communities observed in all uploaded networks, wherein nodes of interest can be searched, highlighted, and visualized in the main network as well. Cliques are also visualized using the same method(s), (g–k) represent visualization of the community of interest in various networks. In panel 2, figure (a) represents visualization of network graph in various layouts offered by NetConfer, (b) depicts the union visualization method adopted by NetConfer using pie-nodes and multi-colored edges, and (c) represents a radar chart showing relative distances of a set of selected networks from their union network

A flowchart of all the steps associated with requirements and submission of a task/job to NetConfer is summarized in Fig. 2. Figure 3 provides a gallery of important visualizations demonstrating the various outputs of NetConfer.

### Details of backend methodology

#### Network similarity calculation

NetConfer utilizes the edge similarity using Jaccard distance [26] that calculates the ratio of intersecting edges (between the compared pair) over their union. The all versus all edge distance is first calculated in a matrix which is then hierarchically clustered to generate a dendrogram available in the Python networkx module (https://networkx.github.io). A hierarchy of the individual networks is built by progressively merging clusters obtained using the pairwise distance measures. The resultant tree is displayed in the main “Workflow Dashboard” with each leaf as a checkbox. In addition to the edge Jaccard, the node Jaccard distance is also calculated and used to calculate the distance of a set of individual networks from their union. The output is displayed as a radar plot available in the “Visualization Dashboard.”

#### Shortest path calculation

Shortest path is the set of minimum edges required to connect a given “source” to a “target” node in a given network. It can be noted that multiple shortest paths can exist between one “source” and “target” node with some nodes serving as preferred intermediates. NetConfer uses Dijkstra’s algorithm [27] implementation in the Python networkx module for this purpose. The algorithm works by generating a shortest path tree with the source node as root and proceeds using two sets, one containing a track of the nodes used in the shortest path and remaining nodes in the other. In every step, a vertex from the other set having the least distance from the source is identified and added to the path. For multiple input networks, NetConfer stores all the path information and displays them together as a path matrix. The layout of all shortest paths is designed in such a manner that the user-specified source and target nodes are positioned at the bottom and top of the path (respectively), and all other nodes between them are numbered in the order they appear in the path (starting from source, which is assigned the order number 0). Consequently, the number on the top (pertaining to the target node) also indicates the total path length.

#### Community detection

NetConfer implements the “fast modularity maximization algorithm” [28] to identify communities in an input network. This algorithm has been proven to work efficiently even with larger input networks. We used the well-known SNAP library [19] for community detection. The modified C++ implementation from the SNAP library and in-house python codes were used in our platform to enable batch calculations. The same library is also used for calculating various graph properties in NetConfer. As opposed to other implementations, this community detection algorithm works using a greedy optimization on the modularity using sophisticated data structures. A dendrogram of hierarchically decomposed communities is first created with leaves as the vertices of the original network and internal nodes representing the joins. The algorithm minimizes needless operations in storing the original data matrix and achieves a dramatic improvement in speed when compared to other implementations. A more detailed and technical description of the algorithm is provided by the authors in the original publication [28].

#### Community transitions

The identified communities can be compared for their similarities in the constituent nodes between a pair of networks. Each community in one network may split into multiple communities in the other network. In order to quantify this “community transition,” an individual Jaccard score as well as intersection count is first calculated across all communities between the two networks and presented as a tabulated summary under “community transitions” tab in “workflow 4.” Two other scores, namely sum Jaccard and weighted sum Jaccard, are also calculated to quantify the overall transition process. As the name implies, the sum Jaccard score is a cumulative sum of all the individual Jaccard scores, while its weighted version is calculated by dividing the value with total community comparisons made and multiplying the result with 100. Given that the Jaccard score calculates the “intersection over union” values, it is imperative that a higher value would indicate a higher intersection and hence higher similarity. Hence, a lower weighted Jaccard score indicates a higher amount of community transitions.

#### Clique finding

Similar to the community detection implementation, NetConfer implements the C++ function of clique finding available in the SNAP library (https://snap.stanford.edu/). The function enumerates maximum cliques within a reasonable time for a given network. The clique finding problem refers to finding the complete subgraphs of a given size (usually denoted as “k”) and subsequently finding whether any other cliques of higher size exist in the input graph. When a user sets the “k” value in the “workflow 5,” all cliques of size ≥ *k* are calculated and displayed as a table. The tabulated result is stored using an efficient JavaScript data structure called “DataTables” (https://datatables.net) in order to perform quick searches with multiple filters.

## Results

### Case study 1: Understanding the role of gut microbiome in multiple sclerosis using microbial association networks

In order to demonstrate the utility of NetConfer tool, we chose a publicly available dataset [29] pertaining to gut microbiomes from 60 multiple sclerosis patients spanning three sets (28 without any treatment, 18 treated with interferon, and 14 treated with Copaxone for at least 6 months) and 43 control subjects. The microbial abundance data pertaining to the study was downloaded from MGnify [30] corresponding to the study ID MGYS00001194. Further, microbial association networks (at genera level) corresponding to all the four groups (“Control,” “Untreated,” “Interferon,” and “Copaxone”) were generated using the abundance data using the CCREPE tool [31] with significant correlations (*p* < 0.005) at 1000 bootstrap iterations. The global graph properties of the four networks indicated the disease groups (both treated and untreated) to have little lower number of edges as compared to the control (Additional file 3: Fig. S21). The “network tree” feature in NetConfer interestingly showed grouping of the “control” and “untreated” samples together while the two treatment networks clustered far apart (Additional file 3: Fig. S22). The “edge upset plot” and the “edge Venn diagram,” available under “workflow 1,” further confirmed that the “control” and “untreated” network did share the highest number of edges compared to others (Additional file 3: Fig. S23,S24). The edge Venn diagram also showed a fair share of exclusive edges in each network indicating some amount of network rewiring. The observations suggested an overall microbiome community structure change in the treatment group. In order to further investigate the changes pertaining to the key members (or important nodes) of the networks, the degree of each node was visualized using the NetConfer’s network view option (by clicking the individual network names or labels from the checkboxes) of the generated tree. The obtained network layout, with node sizes mapped to degree centrality and high degree nodes placed in the center, provided some additional insights (Additional file 3: Fig. S25). While microbial genera like *Enterococcus*, *Clostridium*, *Streptococcus*, and *Actinomyces* were seen as high degree nodes in the “control” network, genera like *Aggregatibacter*, *Staphylococcus*, *Cronobacter*, *Gemella*, *Corynebacterium*, *Ruminococcus*, and *Turicibacter* were found to be prominent key members in the remaining three networks. In order to track how the central nodes in the “control” network changed in the “disease” and “treatments,” NetConfer’s “workflow 2” was used to identify the top 10 high degree nodes in the “control” network and to visualize the changes in their centrality values. As seen earlier in the analysis, the key nodes also appeared to be consistent in their degree in the “control” and “untreated” networks and underwent a major change in the treated samples, the most prominent change being in the interferon-treated patients (Additional file 3: Fig. S26). None of the genera in the top 10 degree of “control” as well as “untreated” was found to be common with the differentially abundant genera reported in the original study. This observation is not surprising considering the fact that a microbial association network captures the similarity changes of intermicrobial associations rather than their individual abundance level changes. Since pathogenic genera like *Staphylococcus*, *Streptococcus*, and *Enterococcus* were observed among the top 10 high degree nodes, we evaluated their colonization as well as communication patterns across the networks using NetConfer. Results obtained using the shortest path workflow indicated that the direct communication between *Enterococcus* and *Streptococcus*/*Staphylococcus* was interrupted during the Copaxone treatment, the minimum path length being found to be increased to 2 (Additional file 3: Fig. S27). However, the number of shortest paths connecting the genera was seen to increase, which might be an indicator of the pathogens trying to find alternate means of communication using other genera as intermediate. The results of the clique analysis (in “workflow 5”) for identifying the “partners in crime” for the above pathogenic genera indicated genera like *Granulicatella*, *Actinomyces*, and *Gemella* to be the likely players in pathogen colonization (Additional file 3: Fig. S28,S29). The visual outputs of the comparison of microbial community structures under different conditions, obtained using workflow 5 of NetConfer, indicated a drastic community change in the interferon treatment (weighted Jaccard score = 1.87), followed by the Copaxone treatment (weighted Jaccard score = 3.03) with respect to the untreated as compared to the untreated with respect to control. As discussed earlier in the “Methods” section, it must be noted that a lower “weighted Jaccard score” indicates higher community change. The networks between the control and untreated group showed an overall lesser change (weighted Jaccard score = 3.8) in their community structure as compared to the changes observed after treatment (Additional file 3: Fig. S30). The observations from the microbiome networks clearly indicated that the microbiome is more affected during the treatment as compared to the disease itself. Evaluating the effectiveness of interferon versus Copaxone is an active area of research [32], and the microbiome might serve as a valuable component to enhance this understanding.

### Case study 2: Understanding differential mycobacterium infection using time series gene networks

The mechanism of *Mycobacterium tuberculosis* infection in human host provides a ground for interesting clinical research [33]. For a long time, researchers have tried to understand the differences in human gene perturbation upon infection by virulent (H37Rv) and avirulent (H37Ra) *M. tuberculosis* strains. In one such effort, a gene expression study on human macrophages infected with H37Ra and H37Rv at different infection time points was performed [23]. The gene expression data obtained from the study (https://www.jbc.org/content/suppl/2011/09/26/M111.266239.DC1/jbc.M111.266239-2.xls) was later analyzed using various bioinformatics approaches including gene perturbation networks [8] by overlaying the highly perturbed genes at different time points on the STRING human reference network [34]. In this case study, we used the above gene perturbation networks for the three infection time points (16 h, 48 h, and 90 h) corresponding to each strain (totalling to 6 networks) to demonstrate the applicability of NetConfer features. The networks, available as edge lists of gene IDs, were uploaded to the NetConfer web server, and the summary global property plot was obtained (Additional file 4: Fig. S31) which showed an increased gene perturbation at the 48 h and 90 h time points for H37Ra as evident from the higher node and edge counts. However, the density of the H37Rv networks showed a higher trend as compared to that of its counterpart H37Ra. The clustered network tree (Additional file 4: Fig. S32A) generated in the next step also showed a distinct cluster of the post-infection time points (for both strains) with the 90th hour time points showing a slightly more difference. This trend was also more pronounced for the H37Ra 90 h network observed in the radar plot (Additional file 4: Fig. S32B) which plots the Jaccard node and edge distance of each network from the union network of the selected set. An inference of the network similarities using the Venn diagram (available under “workflow 1”) could identify and list the contributing nodes representing the gene names (Additional file 4: Fig. S33). High betweenness nodes (genes) for H37Rv 90 h network could be identified using “workflow 2” and compared for their changes across other networks/properties (Additional file 4: Fig. S34A, B). The “BIRC5” node, a gene known to be an inhibitor of apoptosis [35], was found to have highest betweenness value in the H37Rv 90 h network. Interestingly, when the value was compared across other networks, it was found to be the least even with respect to the H37Rv 48 h network. The clique analysis for the virulent time points using “workflow 5” also showed higher size cliques forming only in the 48th hour time point, indicating major connected perturbation events taking place at this time (Additional file 4: Fig. S35). In order to get an idea of the connected perturbation events, we selected another important cell division gene “ASPM” [36] from the list of high betweenness nodes as seen in Additional file 4: Fig. S34. The result showed a list of differential shortest paths taken in H37Ra and H37Rv strains for the different time points (Additional file 4: Fig. S36). The gene “NCAPG” which encodes for proteins responsible for stabilizing the chromosome during cell division [37] forms an important connecting member for all the paths. The H37Rv 90th hour time point showed an exclusive shortest path using the “CCNB1” gene which is known to encode important protein for cell cycle transition phase [38]. An improved understanding of this transition can be visualized using the “community transition plot” available in “workflow 4” of NetConfer (Additional file 4: Fig. S37).

## Discussion

In order to perform a comparative evaluation of execution times for computationally expensive modules in NetConfer, random networks of varied sizes were created. This random network sets consisted of seven categories with total nodes ranging from 500 to 5000 and total edges between 1000 and 25,000. Each category was designed to contain networks of “n” nodes having “2n,” “3n,” “4n,” and “5n” edges, respectively. Further, three replicates were created for each type of network (having same nodes and edge counts, but having varied types of edge connections) and were uploaded to the NetConfer web server for evaluation. This evaluation included four computationally exhaustive operations, namely, network loading and global property calculation, local graph property calculations, community prediction, and clique finding. The results indicated that the maximum time taken for an operation for calculating local graph properties for the biggest network set consisting of 5000 nodes and 25,000 edges was 134.81 s. The maximum time taken for community and clique finding of this biggest set was also less than a minute. A detail of the evaluation for all the sets is provided in Additional file 5: Table 2. Networks of up to 10,000 and 20,000 nodes were found to get uploaded and processed for global properties in less than 20 s and 40 s per network, respectively. Time taken for centrality measure assessment for all networks was observed to be less than a minute and 3 min per network for networks of aforementioned sizes. Other available workflows were processed in less than 3 s per network.

The primary intention of the case studies was to illustrate the ease with which complex comparative analysis of biological networks can be performed using NetConfer. Our tool offers the unique feature of segregating important biological analyses into different workflows which can be easily used by researchers. The case studies provide a demonstrative analysis for each workflow that might serve as a practical guide where it can be applied. The results presented in the case studies however might require addition experimental analysis and validation.

## Conclusions

Given the established importance of interpreting biological information using networks, there is also an inherent need for tools which can compare and visualize multiple networks. The general intention behind developing NetConfer is to ease the accessibility and comprehension bottlenecks often faced by not only biologists or clinicians, but also users looking for quick and easy way of comparing multiple networks. Our tool is expected to be of particular use for smaller or mid-sized networks (nodes < 1000) which are very common in biology. Examples include pathway networks, microbiome genera interactions, residue interaction networks (RINs), and connected gene perturbations. Researchers might also be interested to trim a protein-protein interaction (PPI) network to include only the most significant edges and perform quick comparisons. NetConfer is expected to fill the existing gaps in comparative network biology with pre-defined, logically connected, easy to use workflows and modules. Each workflow is developed keeping a biological analysis objective in mind which is demonstrated using examples and real-world case studies. The NetConfer web version is tested to work efficiently with networks containing total nodes up to 5000 and total edges up to 25,000. Larger networks consisting of up to 20,000 nodes and 40,000 edges are also processed for analyzing network properties, Jaccard similarity assessment, set based comparisons (through Venn diagrams), shortest path analysis, and radar plot generation. However, the stand-alone codes corresponding to each of the NetConfer workflow (accessible through the project page under “offline implementation” section in the footer) can process even larger networks in a local/offline desktop containing the required libraries/software along with relevant documentation. Based on user suggestions, NetConfer will also undergo continuous development and updates to accommodate as many features as possible. We expect our tool to be a valuable contribution in the field of network analysis and comparison.

## Supplementary information

**Additional file 1: Table 1.** Comparison of NetConfer features with other tools.

**Additional file 2: Fig. S1-S20.** List of figures demonstrating various functionalities of NetConfer.

**Additional file 3: Fig. S21-S30.** List of figures corresponding to ‘case study 1’ as described in the manuscript.

**Additional file 4: Fig. S31-S37.** List of figures corresponding to ‘case study 2’ as described in the manuscript.

**Additional file 5: Table 2.** Time evaluation of computationally intensive modules in NetConfer.

## Data Availability

All data generated or analyzed during this study are included in this published article and its supplementary information files. Project name: NetConfer Project home page: https://web.rniapps.net/netconfer Offline implementation: Provided under “offline implementation” section in the footer of the Project homepage Operating system(s): Platform independent Programming language: Python, JavaScript, PHP and HTML Other requirements: A modern browser License: Free for academic use Any restrictions to use by non-academics: Commercial users please contact sharmila.mande@tcs.com

## References

[1] Kuntal BK, Chandrakar P, Sadhu S, Mande SS (2019). “NetShift”: a methodology for understanding “driver microbes” from healthy and disease microbiome datasets. ISME J.

[2] Zheng G, Huang T (1754). The reconstruction and analysis of gene regulatory networks. Methods Mol Biol.

[3] Faust K, Lima-Mendez G, Lerat J-S, Sathirapongsasuti JF, Knight R, Huttenhower C, et al. Cross-biome comparison of microbial association networks. Front Microbiol. 2015;6. 10.3389/fmicb.2015.01200. 10.3389/fmicb.2015.01200 PMC4621437 26579106

[4] Pavlopoulos GA, Secrier M, Moschopoulos CN, Soldatos TG, Kossida S, Aerts J (2011). Using graph theory to analyze biological networks. BioData Min.

[5] Shannon P, Markiel A, Ozier O, Baliga NS, Wang JT, Ramage D (2003). Cytoscape: a software environment for integrated models of biomolecular interaction networks. Genome Res.

[6] Cowley MJ, Pinese M, Kassahn KS, Waddell N, Pearson JV, Grimmond SM (2012). PINA v2.0: mining interactome modules. Nucleic Acids Res..

[7] Goenawan IH, Bryan K, Lynn DJ (2016). DyNet: visualization and analysis of dynamic molecular interaction networks. Bioinformatics..

[8] Kuntal BK, Dutta A, Mande SS. CompNet: a GUI based tool for comparison of multiple biological interaction networks. BMC Bioinformatics. 2016;17. 10.1186/s12859-016-1013-x. 10.1186/s12859-016-1013-x PMC4845442 27112575

[9] Yip KY, Yu H, Kim PM, Schultz M, Gerstein M (2006). The tYNA platform for comparative interactomics: a web tool for managing, comparing and mining multiple networks. Bioinformatics..

[10] Brohée S, Faust K, Lima-Mendez G, Sand O, Janky R, Vanderstocken G (2008). NeAT: a toolbox for the analysis of biological networks, clusters, classes and pathways. Nucleic Acids Res..

[11] Zhou G, Xia J (2018). OmicsNet: a web-based tool for creation and visual analysis of biological networks in 3D space. Nucleic Acids Res.

[12] Gerasch A, Faber D, Küntzer J, Niermann P, Kohlbacher O, Lenhof H-P (2014). BiNA: a visual analytics tool for biological network data. PLoS One.

[13] Theodosiou T, Efstathiou G, Papanikolaou N, Kyrpides NC, Bagos PG, Iliopoulos I (2017). NAP: the Network Analysis Profiler, a web tool for easier topological analysis and comparison of medium-scale biological networks. BMC Res Notes.

[14] Salavert F, García-Alonso L, Sánchez R, Alonso R, Bleda M, Medina I (2016). Web-based network analysis and visualization using CellMaps. Bioinformatics..

[15] Bharadwaj A, Singh DP, Ritz A, Tegge AN, Poirel CL, Kraikivski P (2017). GraphSpace: stimulating interdisciplinary collaborations in network biology. Bioinformatics..

[16] Zhou G, Soufan O, Ewald J, Hancock REW, Basu N, Xia J (2019). NetworkAnalyst 3.0: a visual analytics platform for comprehensive gene expression profiling and meta-analysis. Nucleic Acids Res.

[17] Wang Y, Thilmony R, Gu YQ (2014). NetVenn: an integrated network analysis web platform for gene lists. Nucleic Acids Res..

[18] Franz M, Lopes CT, Huck G, Dong Y, Sumer O, Bader GD (2016). Cytoscape.js: a graph theory library for visualisation and analysis. Bioinformatics..

[19] Leskovec Jure, Sosič Rok (2016). SNAP. ACM Transactions on Intelligent Systems and Technology.

[20] Bardou Philippe, Mariette Jérôme, Escudié Frédéric, Djemiel Christophe, Klopp Christophe (2014). jvenn: an interactive Venn diagram viewer. BMC Bioinformatics.

[21] Lex A, Gehlenborg N, Strobelt H, Vuillemot R, Pfister H (2014). UpSet: visualization of intersecting sets. IEEE Trans Vis Comput Graph.

[22] Bhatt V, Mohapatra A, Anand S, Kuntal BK, Mande SS. FLIM-MAP: gene context based identification of functional modules in bacterial metabolic pathways. Front Microbiol. 2018;9. 10.3389/fmicb.2018.02183. 10.3389/fmicb.2018.02183 PMC6157337 30283416

[23] Karim AF, Chandra P, Chopra A, Siddiqui Z, Bhaskar A, Singh A (2011). Express path analysis identifies a tyrosine kinase Src-centric network regulating divergent host responses to Mycobacterium tuberculosis infection. J Biol Chem.

[24] Gitter A, Carmi M, Barkai N, Bar-Joseph Z (2013). Linking the signaling cascades and dynamic regulatory networks controlling stress responses. Genome Res.

[25] Pradhan MP, Nagulapalli K, Palakal MJ (2012). Cliques for the identification of gene signatures for colorectal cancer across population. BMC Syst Biol..

[26] Jaccard P (1912). The distribution of the flora in the Alpine zone. New Phytol.

[27] Frana PL, Misa TJ (2010). An interview with Edsger W. Dijkstra. Commun ACM.

[28] Clauset A, Newman MEJ, Moore C (2004). Finding community structure in very large networks. Phys Rev E.

[29] Jangi S, Gandhi R, Cox LM, Li N, von Glehn F, Yan R (2016). Alterations of the human gut microbiome in multiple sclerosis. Nat Commun.

[30] Mitchell AL, Almeida A, Beracochea M, Boland M, Burgin J, Cochrane G (2020). MGnify: the microbiome analysis resource in 2020. Nucleic Acids Res.

[31] Faust K, Raes J (2012). Microbial interactions: from networks to models. Nat Rev Microbiol.

[32] Melendez-Torres GJ, Armoiry X, Court R, Patterson J, Kan A, Auguste P (2018). Comparative effectiveness of beta-interferons and glatiramer acetate for relapsing-remitting multiple sclerosis: systematic review and network meta-analysis of trials including recommended dosages. BMC Neurol.

[33] Chai Q, Zhang Y, Liu CH. Mycobacterium tuberculosis: an adaptable pathogen associated with multiple human diseases. Front Cell Infect Microbiol. 2018;8. 10.3389/fcimb.2018.00158. 10.3389/fcimb.2018.00158 PMC5962710 29868514

[34] Szklarczyk D, Franceschini A, Wyder S, Forslund K, Heller D, Huerta-Cepas J (2015). STRING v10: protein–protein interaction networks, integrated over the tree of life. Nucleic Acids Res.

[35] Kelly RJ, Lopez-Chavez A, Citrin D, Janik JE, Morris JC (2011). Impacting tumor cell-fate by targeting the inhibitor of apoptosis protein survivin. Mol Cancer.

[36] Capecchi MR, Pozner A (2015). ASPM regulates symmetric stem cell division by tuning Cyclin E ubiquitination. Nat Commun.

[37] Liu W, Liang B, Liu H, Huang Y, Yin X, Zhou F (2017). Overexpression of non-SMC condensin I complex subunit G serves as a promising prognostic marker and therapeutic target for hepatocellular carcinoma. Int J Mol Med.

[38] Sartor H, Ehlert F, Grzeschik KH, Müller R, Adolph S (1992). Assignment of two human cell cycle genes, CDC25C and CCNB1, to 5q31 and 5q12, respectively. Genomics..

