# Correction to: NetConfer: a web application for comparative analysis of multiple biological networks

**DOI:** 10.1186/s12915-020-00898-x

**Published:** 2020-10-22

**Authors:** Sunil Nagpal, Krishanu Das Baksi, Bhusan K. Kuntal, Sharmila S. Mande

**Affiliations:** 1 Bio-Sciences R&D Division, TCS Research, Tata Consultancy Services Ltd., 54-B Hadapsar Industrial Estate, Pune, 411 013 India; 2 Chemical Engineering and Process Development Division, CSIR-National Chemical Laboratory, Dr. Homi Bhabha Road, Pune, 411 008 India; 3 Academy of Scientific and Innovative Research (AcSIR), Ghaziabad, 201002 India

**Correction to: BMC Biol 18, 53 (2020)**

**https://doi.org/10.1186/s12915-020-00781-9**

Following publication of the original article [1], the authors identified an error in the affiliation list. Affiliation 3 was incorrectly processed as:

- Academy of Scientific and Innovative Research (AcSIR), CSIR-National Chemical Laboratory Campus, Pune 411 008, India.

Affiliation 3 should have been processed as:

- Academy of Scientific and Innovative Research (AcSIR), Ghaziabad 201002, India.

The corrected affiliation list is reflected in this Correction.
