# Supplementary material for: NetConfer: a web application for comparative analysis of multiple biological networks
Source: BMC Biol. 2020 May 19;18:53. doi: 10.1186/s12915-020-00781-9 (PMC7236966; doi:10.1186/s12915-020-00781-9)
Supplement: Supplementary file 1 — Additional file 1: Table 1. Comparison of NetConfer features with other tools. [file 12915_2020_781_MOESM1_ESM.pdf]

Present: green; Absent: red

TOOL	Max input Networks at a time	Web-application	Desktop (UI) application	Command-line application	Compositional assessment	Network property assessment	Shortest Paths	Community composition	Clique analysis	Set comparisons (Venn, Union pie nodes, exclusive etc)	Delta-centrality measures	Inter-network distance measures and visualization	Community transitions and visulization	Clique comparisons and visualization	Shortest path comparison and visualization	Property mapped single network visualization	Brief about the tool	Drawbacks	Advantages of NetConfer over given tool	Reference
**NetConfer**	**8**	Present	Absent	Present	Present	Present	Present	Present	Present	Present	Present	Present	Present	Present	Present	Present	A web-server aimed at analysing, comparing and visualizing multiple biological networks in terms of composition, network properties, shortest paths, community transitions, cliques, network distance measures, etc.	Online version has speed and scale bottlenecks when processing large networks		
**Cytoscape.js**	1	Absent	Absent	Absent	Absent	Present	Absent	Absent	Absent	Absent	Absent	Absent	Absent	Absent	Absent	Absent	A JavaScript library that helps view graphs in different types of layouts, when implemented in a web-application framework.	Main objective visualization. Doesn't have any provision for comparing graphs	The network visualizations in NetConfer are built upon Cytoscape.js, thereby providing more functionality.	Franz et al., 2016
**GraphSpace**	1	Present	Absent	Absent	Present	Present	Absent	Absent	Absent	Absent	Absent	Absent	Absent	Absent	Absent	Present	A web server built specifically to view and share biological networks. Allows users to view networks one at a time. Some basic analysis like finding networks containing a specific node.	Does not have any dedicated network comparison methods. Mainly focuses on network visualization	NetConfer allows users to view the networks as well as compare two or more using different methods.	Bharadwaj et al., 2017
**PINA4MS***	0	Present	Present	Absent	Present	Present	Absent	Absent	Absent	Present	Absent	Absent	Absent	Absent	Absent	Present	Cytoscape plugin that combines protein expression and interaction data for identification of relevant genes.	Specific to requirement of protein-protein interactions pertaining to tissue level niche	NetConfer has been designed such that it can be used for analyzing various types of biological networks, like protein interaction networks, gene networks, microbial networks, etc.	http://apps.cytoscape.org/apps/pina4ms
**DyNet**	100	Absent	Present	Absent	Present	Present	Absent	Absent	Absent	Absent	Absent	Present	Absent	Absent	Absent	Present	Cytoscape plugin that helps comparing networks to identify most rewired nodes.	Specific to the requirement of tracking the rewired nodes	NetConfer is multi-worklfow and multi-modular framework, wherein rewiring tracking is addressed in various workflows like Shortest Paths, Community tracking, Clique changes and more. Apart from this, there are many more useful modules and workflows in NetConfer.	Goenawan et al., 2016
**Venn and Euler Diagrams**	4	Absent	Present	Absent	Present	Absent	Absent	Absent	Absent	Present<br>Only Venn, Euler	Absent	Absent	Absent	Absent	Absent	Absent	Cytoscape plugin useful for generating node composition based Venn and Euler diagrams.	Specific to requirement of compositional comparison only	NetConfer is multi-worklfow and mult-modular framework, wherein compositional comparsions are also covered through Venn, Upset as well as Network graph views in a single workflow. There are many more useful modules and workflows in NetConfer.	https://apps.cytoscape.org/apps/vennandeulerdiagrams
**CellMaps**	1	Present	Absent	Absent	Present	Present	Absent	Present	Absent	Absent	Absent	Absent	Absent	Absent	Absent	Present	Web server specifically designed to do network visualization and analysis, along with options to overlay metadata on the nodes/edges.	No methods/techniques available for multiple network comparison.	NetConfer allows users to view the input networks as well as compare two or more of them using a variety of methods.	Salavert et al., 2016
**NeAT***	0	Absent	Absent	Present	Present	Present	Absent	Absent	Absent	Present	Absent	Absent	Absent	Absent	Absent	Present	Web based tool for network analysis. Several graph algorithms are available in this tool.	This tool has some basic network comparison methods, like finding the union, intersection etc of only two networks.	NetConfer lets users compare more than two networks simultaneously. Also, it provides additional methods for network comparison apart from finding the union/intersection etc.	Brohée et al., 2008
**NAP**<br>combinatorial comparisons not possible	Undefined	Present	Absent	Absent	Present	Present	Absent	Absent	Absent	Present<br>Only intersection (2 at a time)	Present	Absent	Absent	Absent	Absent	Present<br>No property maps	R Shiny based web tool designed to automate network profiling and intra/inter-network topology comparison.	Only a few methods available for multiple network comparison, like network intersection, comparison of topological features of the networks etc.	The workflow 2 in NetConfer incorporates a number of methods for graph comparisons using property measures. Network union and intersection can be easily performed using the visulaization as well as workflow 1.	Theodosiou et al., 2017
**tYNA**<br>combinatorial comparisons not possible	Undefined	Present	Absent	Absent	Present	Present	Absent	Absent	Absent	Present<br>(Only paired comparisons)	Present	Absent	Absent	Absent	Absent	Present	Web based tool for managing, comparing and mining multiple interactomics networks.	Network comparisons limited to intersection and property calculations with basic visualizations.	NetConfer offers users several methods for network comparison, including the ones available in tYNA. Also the tool can be used for any generic biologcal networks, including interactome network.	Yip et al., 2006
**PINA***	0	Present	Present	Absent	Present	Present	Absent	Absent	Absent	Present	Absent	Absent	Absent	Absent	Absent	Present	Web based server and cytoscape app specifically for protein protein interaction networks. Offers users tools for network construction, filtering, analysis and visualization.	Supports network comparison using venn diagram and node pie charts based networks.	NetConfer can be used to compare networks, including but not limited to PPI networks. NetConfer also lets users make Venn diagrams (workflow 1) and Pie Chart based networks (visualization module).	Cowley et al., 2012
**OmicsNet***	0	Present	Absent	Absent	Present	Present	Present	Present	Absent	Absent	Absent	Absent	Absent	Absent	Absent	Present	Web based network creation from multiple lists, visualization and analysis tool.	Main use of this tool pertains to network visualization in 3D space. No methods for network comparison.	NetConfer offers users methods for network comparison along with innovative ways of visualizing the networks and their properties.	Zhou and Xia, 2018

*** Input taken in the form of list of genes or proteins (nodes) rather than network files or edge-lists**

Table 1: Comparison of NetConfer features with other tools
