# Supplementary material for: NetConfer: a web application for comparative analysis of multiple biological networks
Source: BMC Biol. 2020 May 19;18:53. doi: 10.1186/s12915-020-00781-9 (PMC7236966; doi:10.1186/s12915-020-00781-9)
Supplement: Supplementary file 2 — Additional file 2: Fig. S1-S20. List of figures demonstrating various functionalities of NetConfer. [file 12915_2020_781_MOESM2_ESM.pdf]

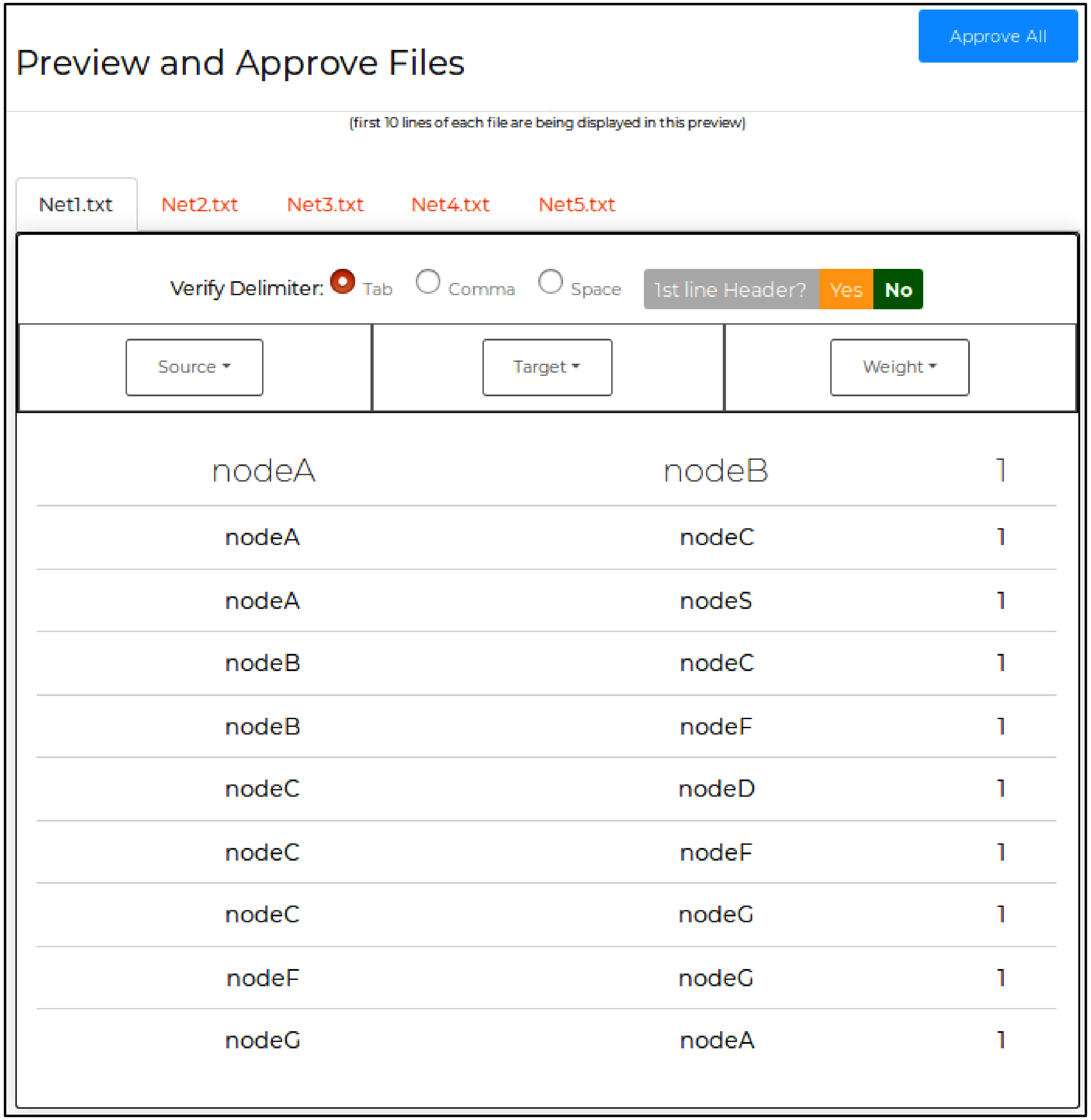


**Figure S1**: Input type (Edge-list)

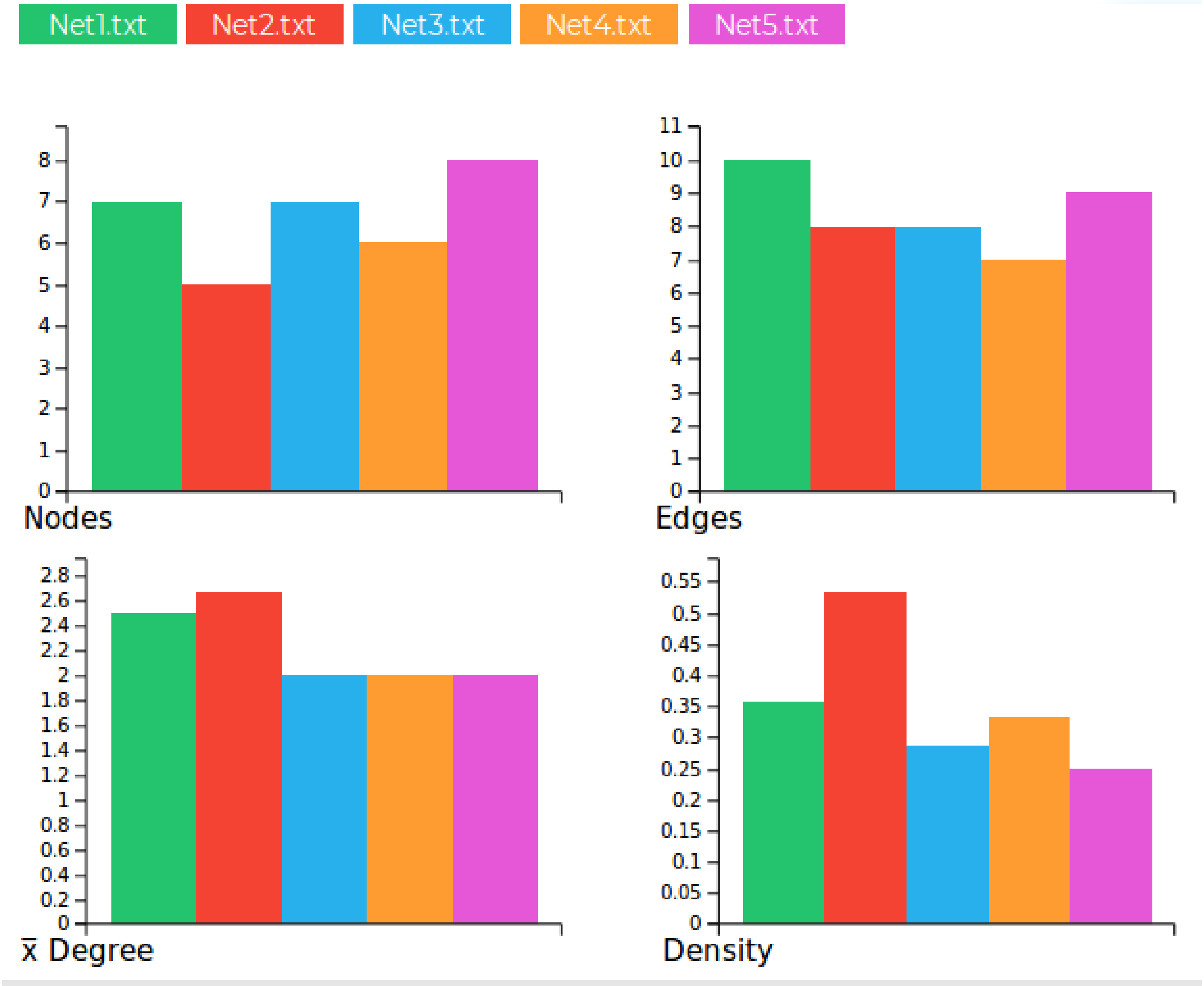


**Figure S2** : Summary of global properties

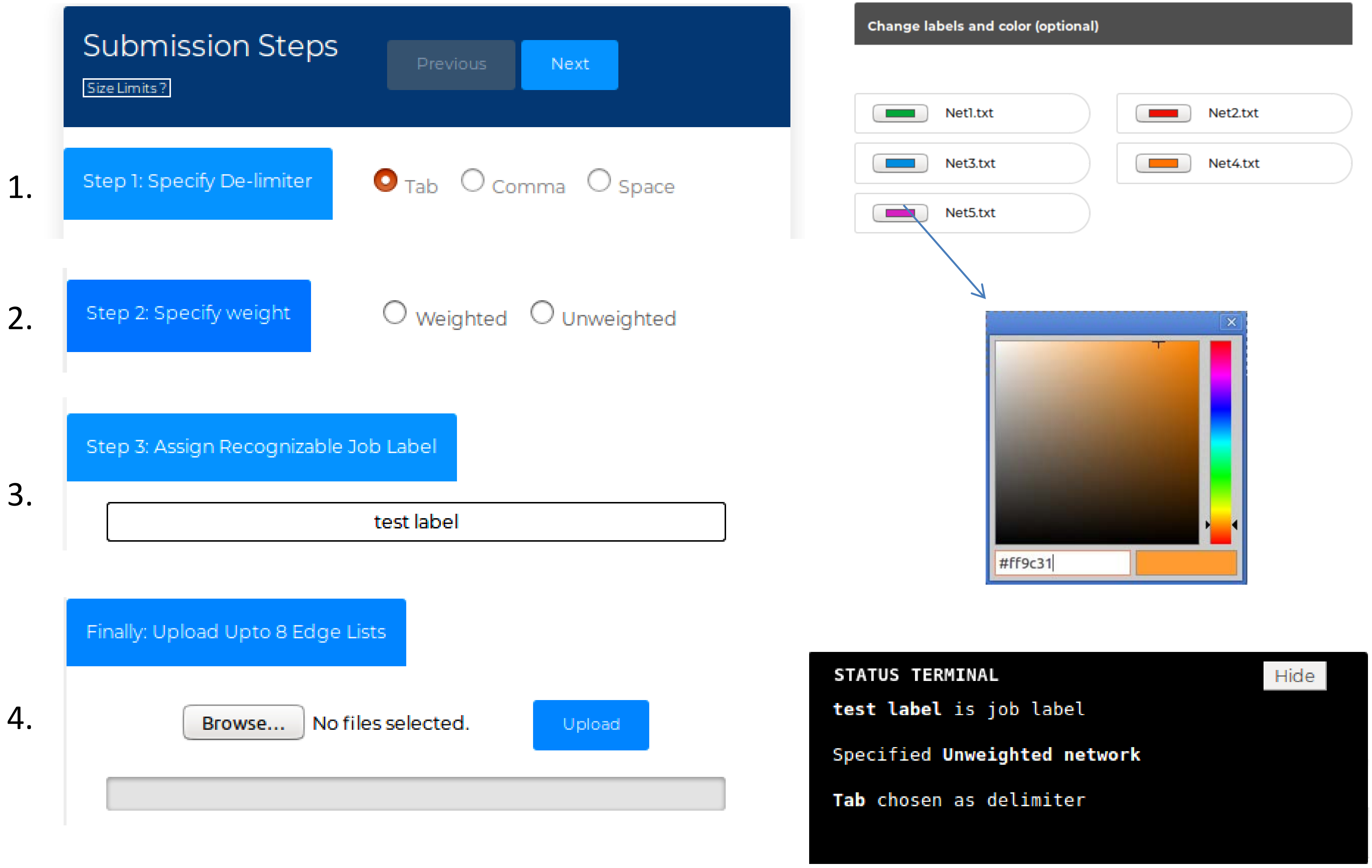


**Figure S3** : Customizing input network names and color

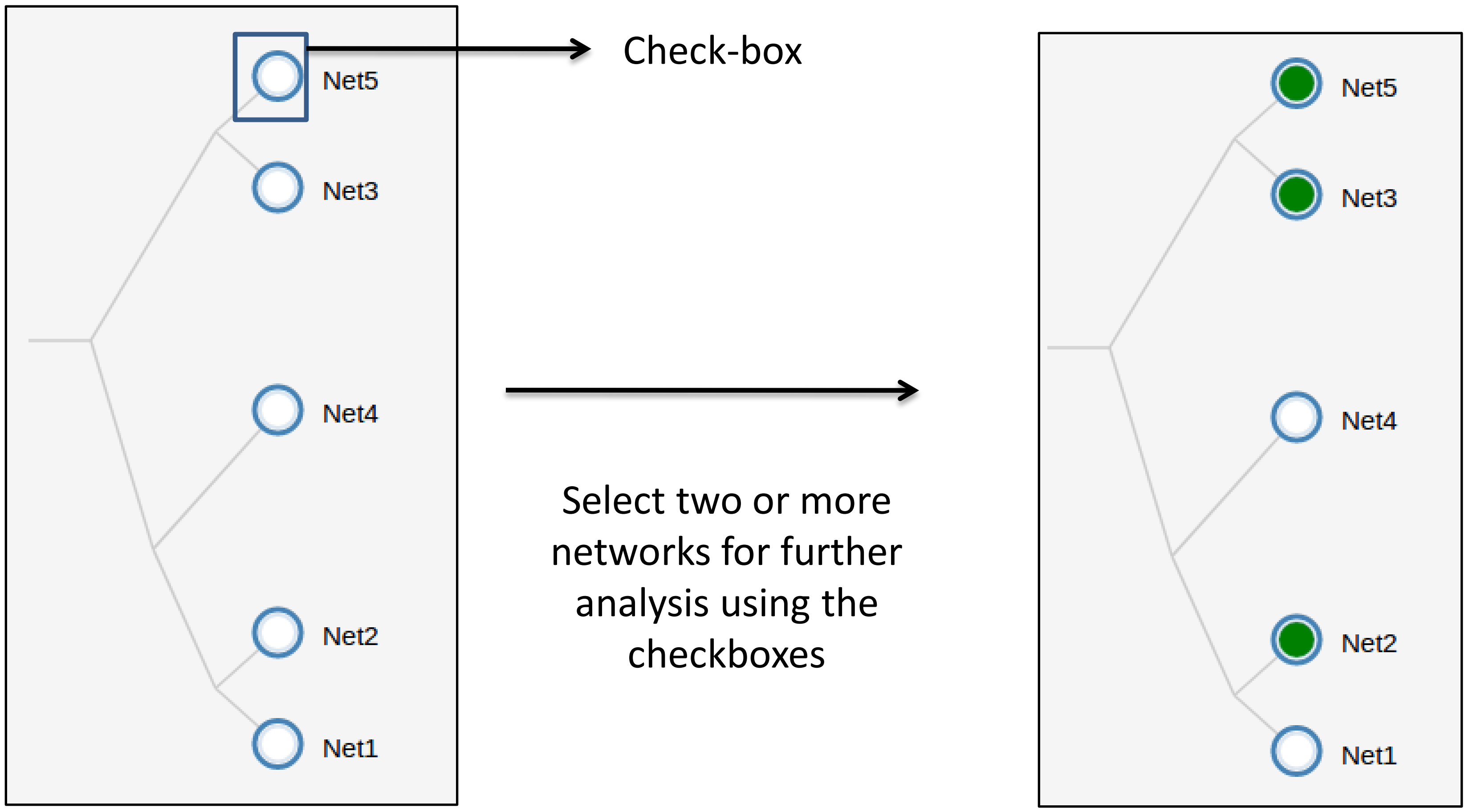


**Figure S4** : Networks auto clustered based on their similarity (Edge-Jaccard score) for ease of selection

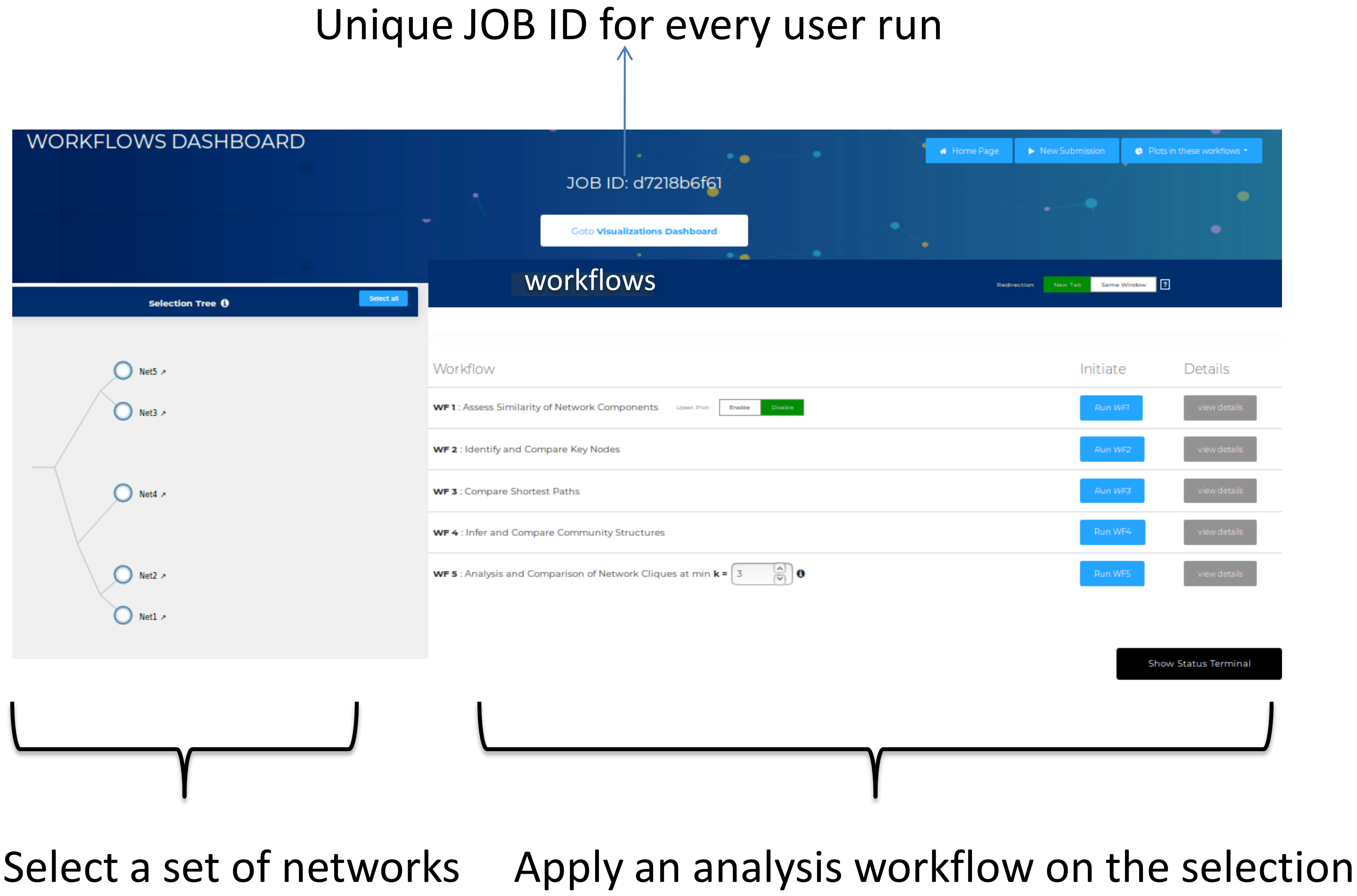


**Figure S5** : The workflow dashboard

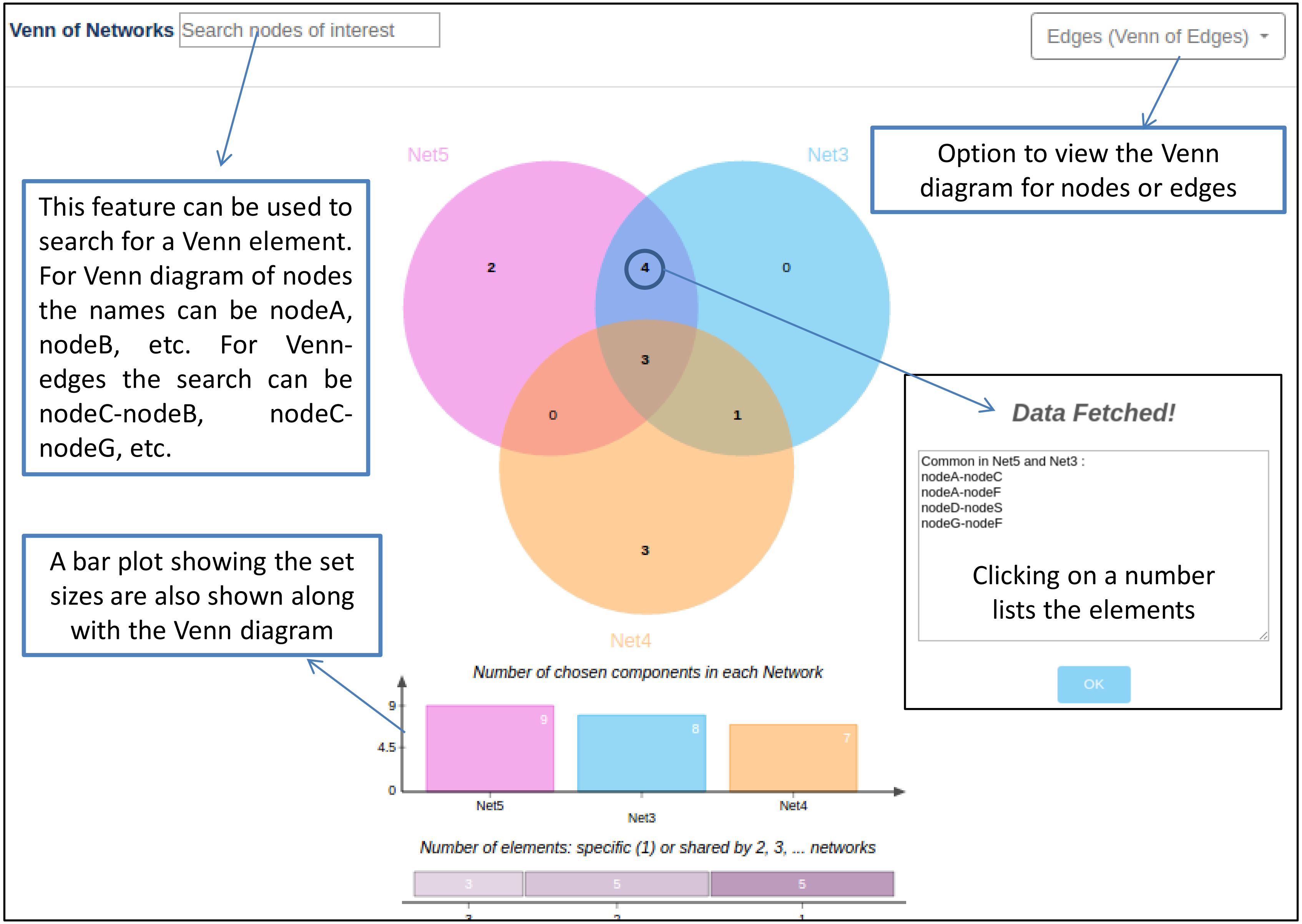


**Figure S6** : Visualize network components using Venn diagrams

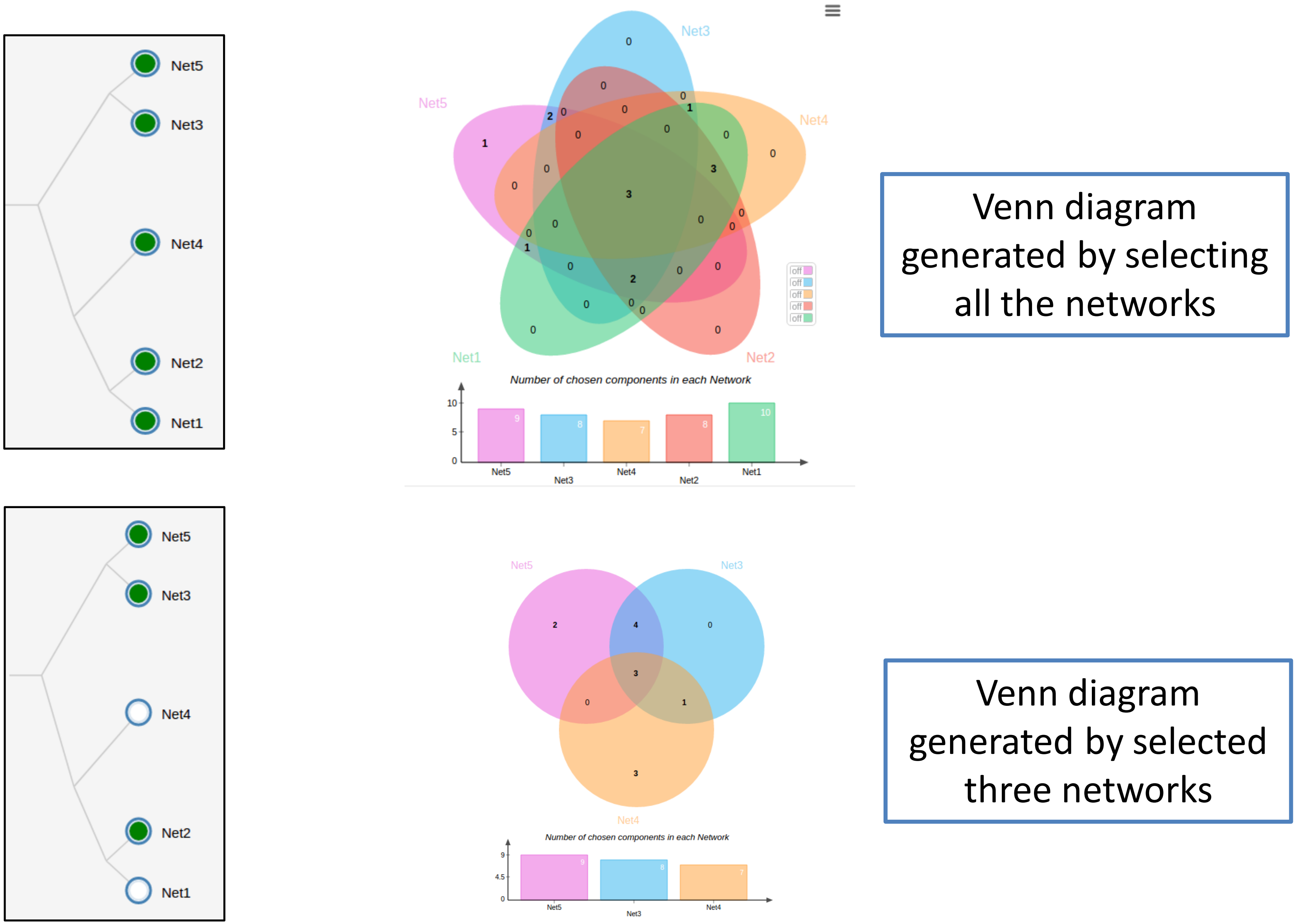


**Figure S7** : Generating Venn diagrams based on user selection

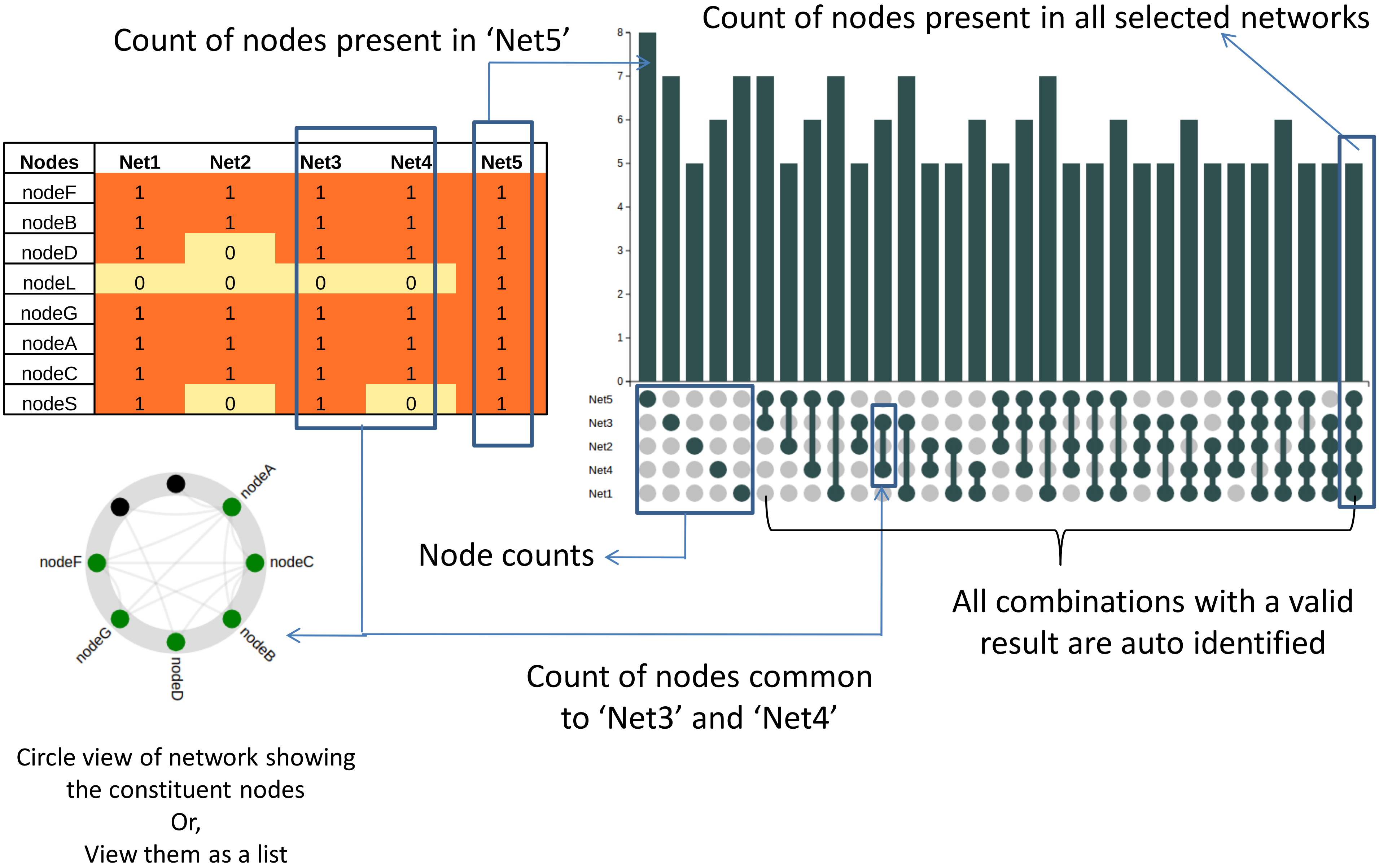


| Nodes | Net1 | Net2 | Net3 | Net4 | Net5 |
|---|---|---|---|---|---|
| nodeF | 1 | 1 | 1 | 1 | 1 |
| nodeB | 1 | 1 | 1 | 1 | 1 |
| nodeD | 1 | 0 | 1 | 1 | 1 |
| nodeL | 0 | 0 | 0 | 0 | 1 |
| nodeG | 1 | 1 | 1 | 1 | 1 |
| nodeA | 1 | 1 | 1 | 1 | 1 |
| nodeC | 1 | 1 | 1 | 1 | 1 |
| nodeS | 1 | 0 | 1 | 0 | 1 |

**Figure S8** : Interpretation of the UpSet plot (Nodes)

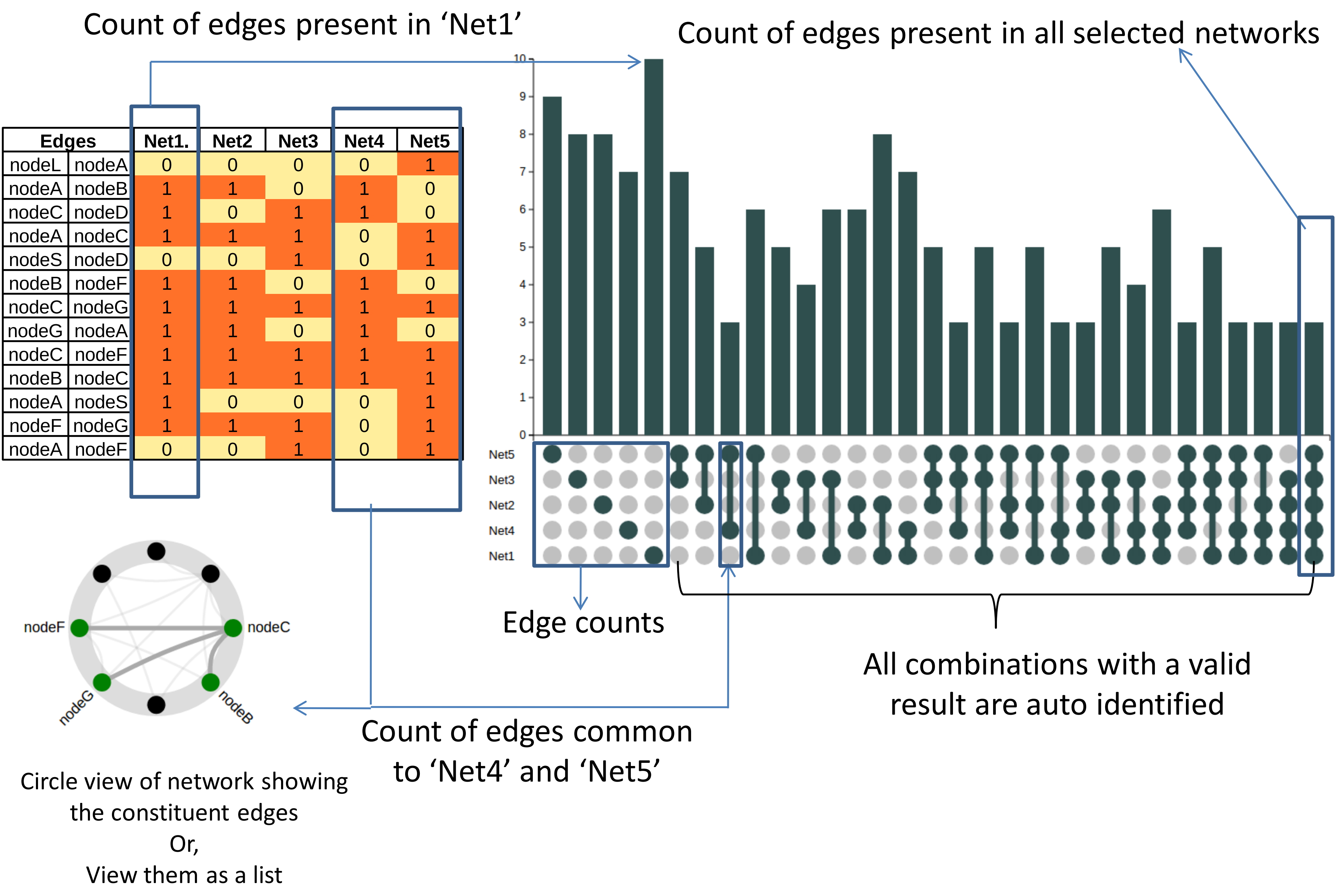


| Edges | | Net1. | Net2 | Net3 | Net4 | Net5 |
|---|---|---|---|---|---|---|
| nodeL | nodeA | 0 | 0 | 0 | 0 | 1 |
| nodeA | nodeB | 1 | 1 | 0 | 1 | 0 |
| nodeC | nodeD | 1 | 0 | 1 | 1 | 0 |
| nodeA | nodeC | 1 | 1 | 1 | 0 | 1 |
| nodeS | nodeD | 0 | 0 | 1 | 0 | 1 |
| nodeB | nodeF | 1 | 1 | 0 | 1 | 0 |
| nodeC | nodeG | 1 | 1 | 1 | 1 | 1 |
| nodeG | nodeA | 1 | 1 | 0 | 1 | 0 |
| nodeC | nodeF | 1 | 1 | 1 | 1 | 1 |
| nodeB | nodeC | 1 | 1 | 1 | 1 | 1 |
| nodeA | nodeS | 1 | 0 | 0 | 0 | 1 |
| nodeF | nodeG | 1 | 1 | 1 | 0 | 1 |
| nodeA | nodeF | 0 | 0 | 1 | 0 | 1 |

**Figure S9** : Interpretation of the UpSet plot (Edges)

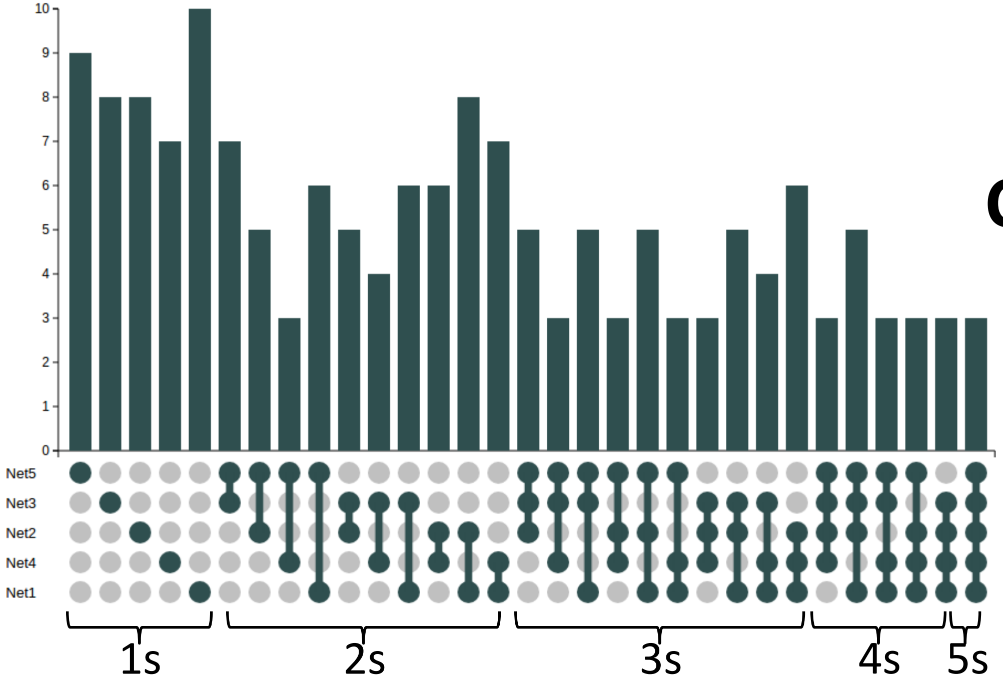


## Combination Cardinality

Plot bars sorted by the size of the combinations

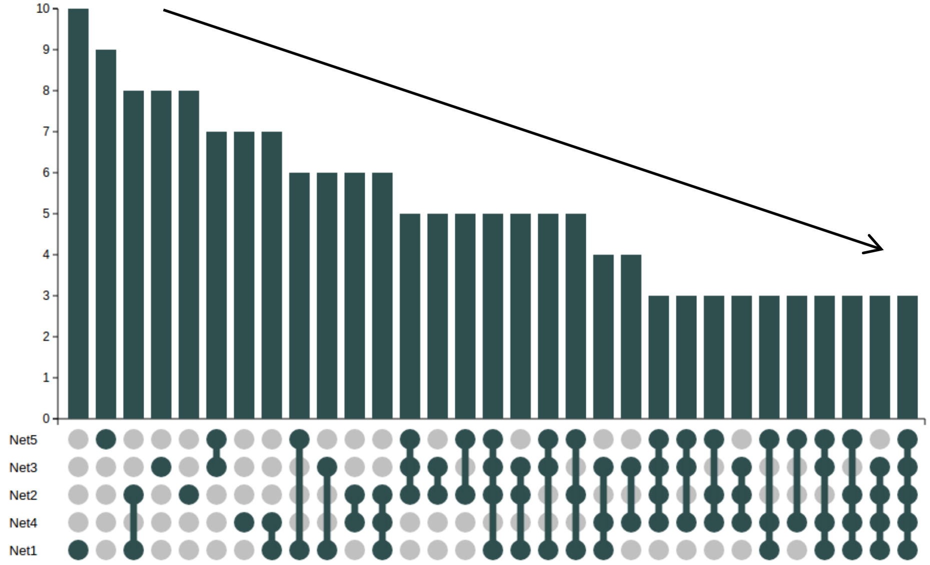


## Set Cardinality

Plot bars sorted by the size of the sets

**Figure S10** : Alternate views for the UpSet plot

Betweenness Degree Closeness HubScore Eigenvector Export Table

**Nodes across networks**

| Nodes | Net5 | Net3 | Net4 | Net2 | Net1 |
|---|---|---|---|---|---|
| nodeA | 14.0 | 0.0 | 0.5 | 0.333 | 5.333 |
| nodeC | 8.0 | 11.5 | 5.5 | 0.667 | 6.0 |
| nodeS | 6.0 | 0.0 | -1.0 | -1.0 | 0.0 |
| nodeF | 2.0 | 0.5 | 0.0 | 0.333 | 0.333 |
| nodeB | 0.0 | 0.0 | 2.0 | 0.333 | 0.667 |
| nodeG | 0.0 | 0.0 | 1.0 | 0.333 | 0.667 |

First Prev 1 2 Next Last

Net5 Net3 Net4 Net2 Net1 Export Table

**Networks across nodes**

| Node | Degree | Closene... | Betwee... | EigenVe... | Network... | Clusteri... | PageRank | HubScore | Authority... |
|---|---|---|---|---|---|---|---|---|---|
| nodeA | 4.0 | 0.7 | 14.0 | 0.489 | 0.375 | 0.167 | 0.111 | 0.806 | 0.0 |
| nodeC | 4.0 | 0.636 | 8.0 | 0.536 | 0.484 | 0.333 | 0.142 | 0.498 | 0.542 |
| nodeS | 2.0 | 0.5 | 6.0 | 0.196 | 0.547 | 0.0 | 0.091 | 0.0 | 0.405 |
| nodeF | 3.0 | 0.583 | 2.0 | 0.487 | 0.708 | 0.667 | 0.151 | 0.168 | 0.655 |
| nodeB | 1.0 | 0.412 | 0.0 | 0.188 | 1.188 | 0.0 | 0.06 | 0.273 | 0.0 |
| nodeG | 2.0 | 0.438 | 0.0 | 0.359 | 0.936 | 1.0 | 0.249 | 0.0 | 0.335 |

First Prev 1 2 Next Last

**Figure S11** : Two way comparison of network properties

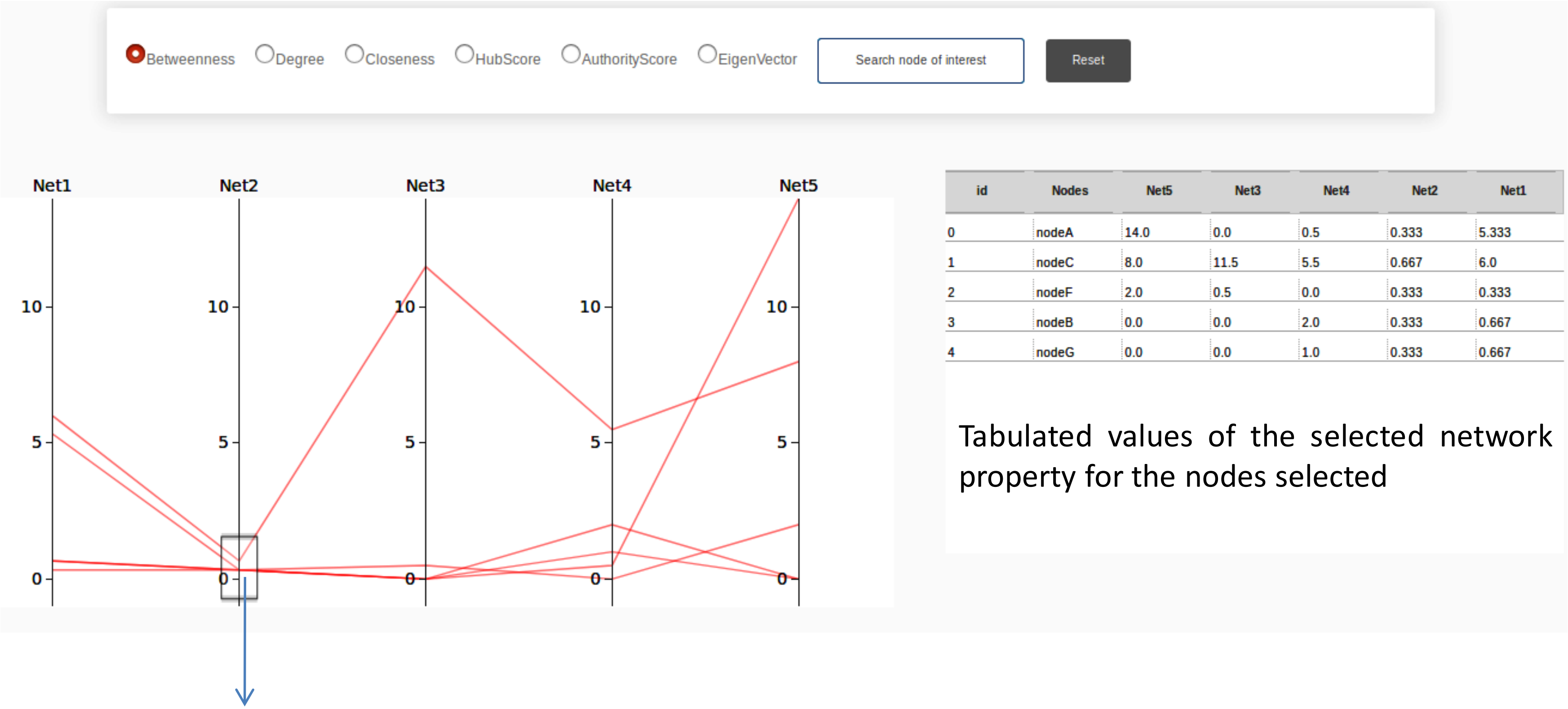


| id | Nodes | Net5 | Net3 | Net4 | Net2 | Net1 |
|---|---|---|---|---|---|---|
| 0 | nodeA | 14.0 | 0.0 | 0.5 | 0.333 | 5.333 |
| 1 | nodeC | 8.0 | 11.5 | 5.5 | 0.667 | 6.0 |
| 2 | nodeF | 2.0 | 0.5 | 0.0 | 0.333 | 0.333 |
| 3 | nodeB | 0.0 | 0.0 | 2.0 | 0.333 | 0.667 |
| 4 | nodeG | 0.0 | 0.0 | 1.0 | 0.333 | 0.667 |

Tabulated values of the selected network property for the nodes selected

Draggable brush selection of nodes

**Figure S12** : Visualize changes in different network properties using parallel coordinates

# Interpretation of the shortest path plot

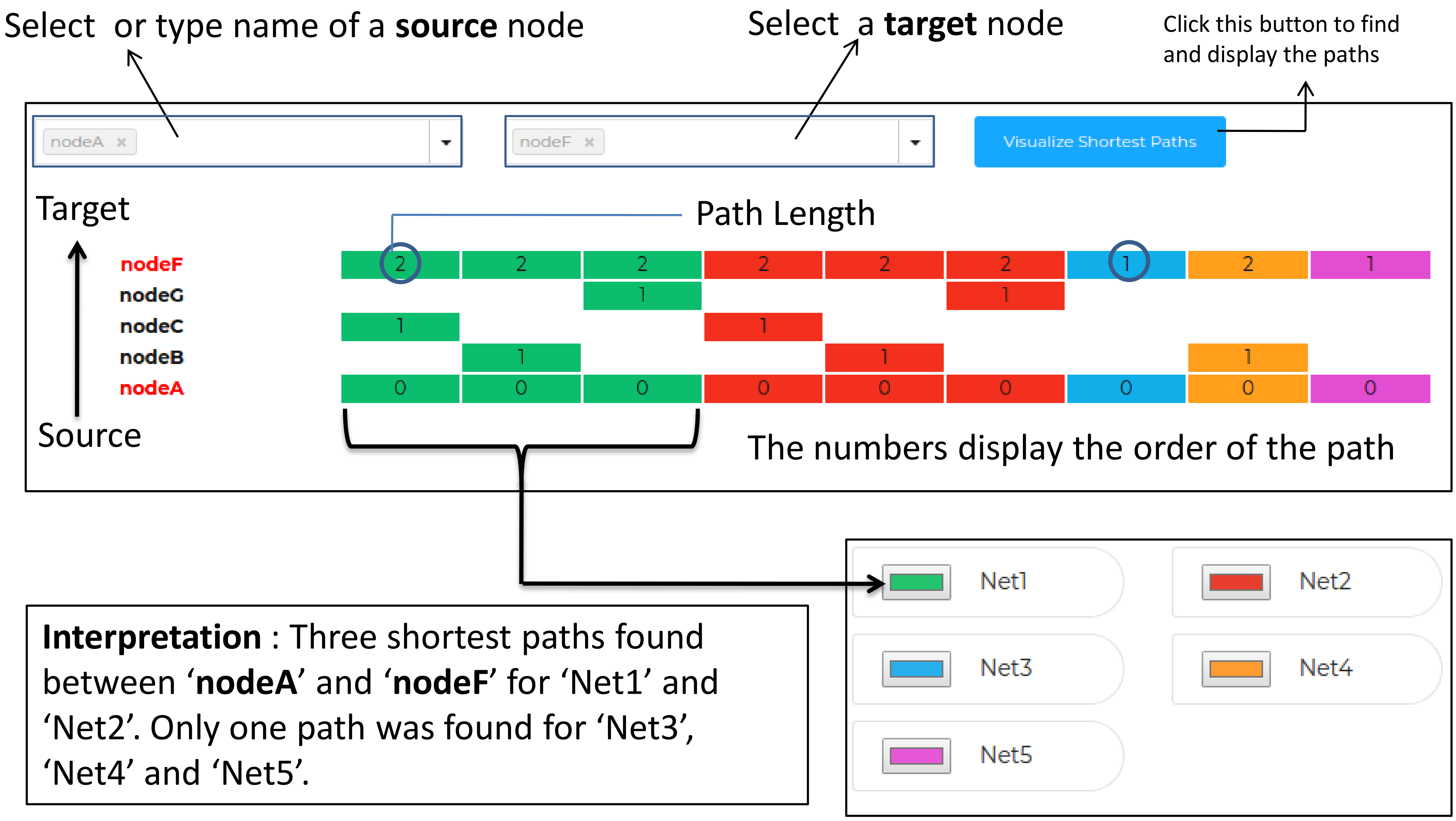


**Figure S13 :** Interpretation of the shortest path plot

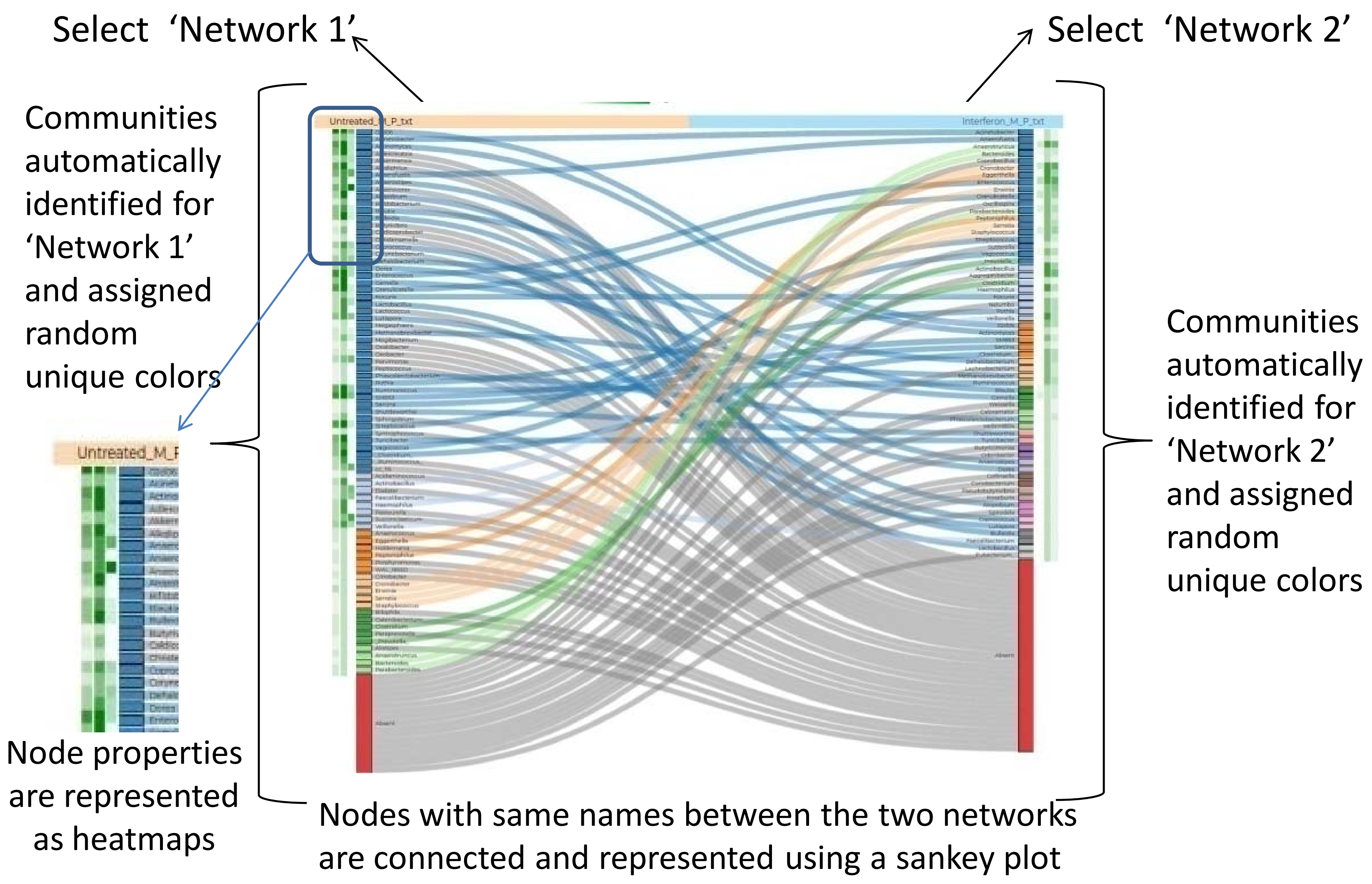


**Figure S14 :** Interpretation of community plot

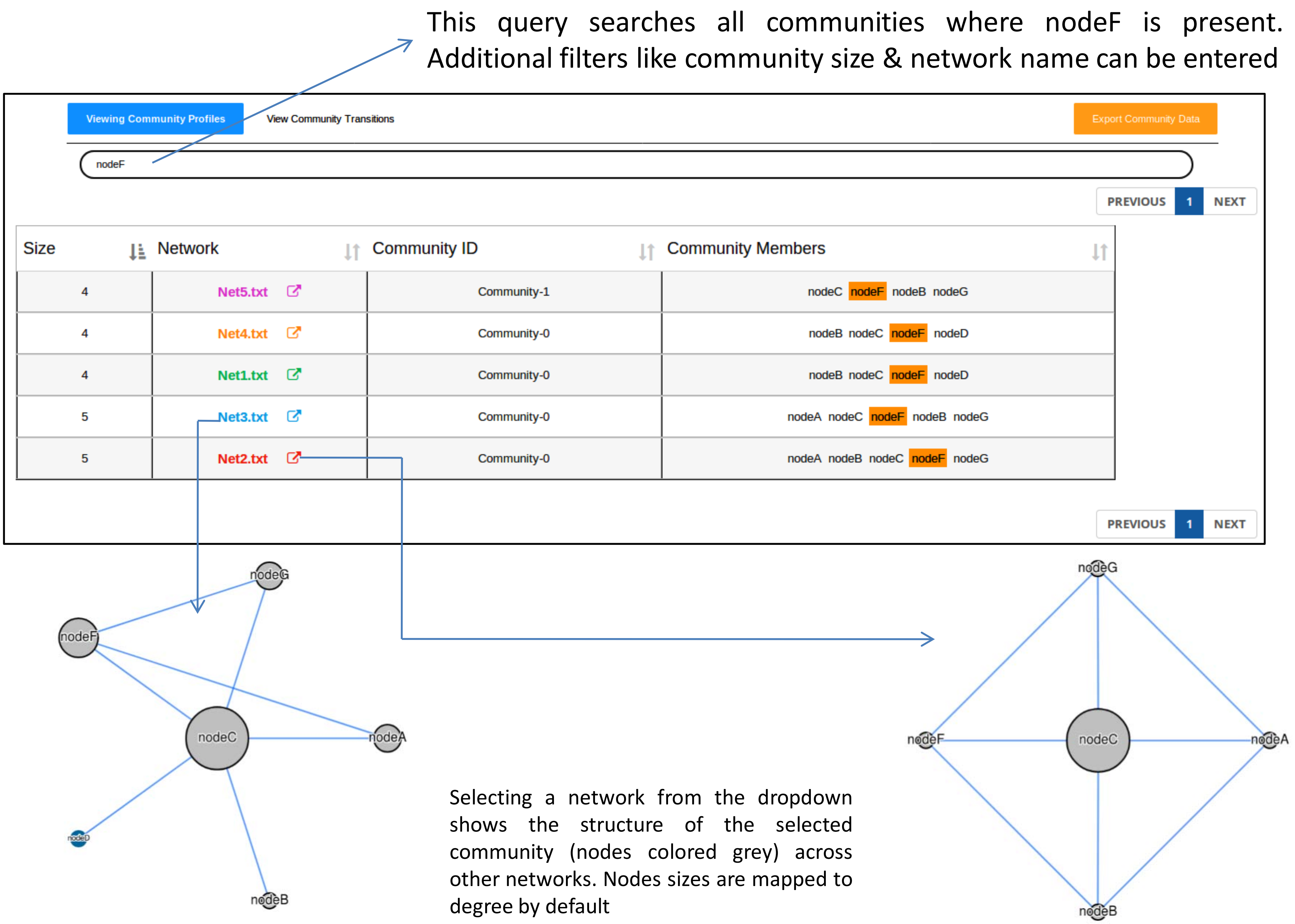


**Figure S15 :** Visualization of the network communities

Search for one or more nodes to list the cliques where they are present across the selected set of networks. The network names can also be added to narrow down the filter criteria.

nodeC nodeG

| Size | Network | CliqueID | Clique Membership |
|---|---|---|---|
| 3 | Net5.txt | Clique-1 | nodeG nodeC nodeF |
| 3 | Net3.txt | Clique-1 | nodeC nodeF nodeG |
| 3 | Net2.txt | Clique-1 | nodeC nodeA nodeG |
| 3 | Net2.txt | Clique-3 | nodeC nodeF nodeG |
| 3 | Net1.txt | Clique-1 | nodeC nodeA nodeG |
| 3 | Net1.txt | Clique-3 | nodeC nodeF nodeG |

- Clicking on the network names displays the network with the clique highlighted in grey
- Similar to communities, the clique structure can be viewed across all the networks
- Cliques are displayed along with their first interacting neighbor nodes highlighted in blue

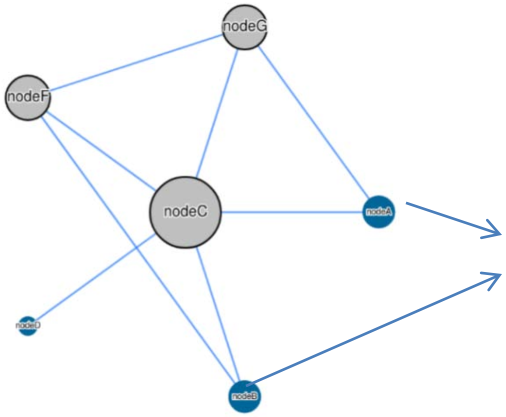


First neighbor nodes of the clique

**Figure S16 :** Visualization of the network cliques

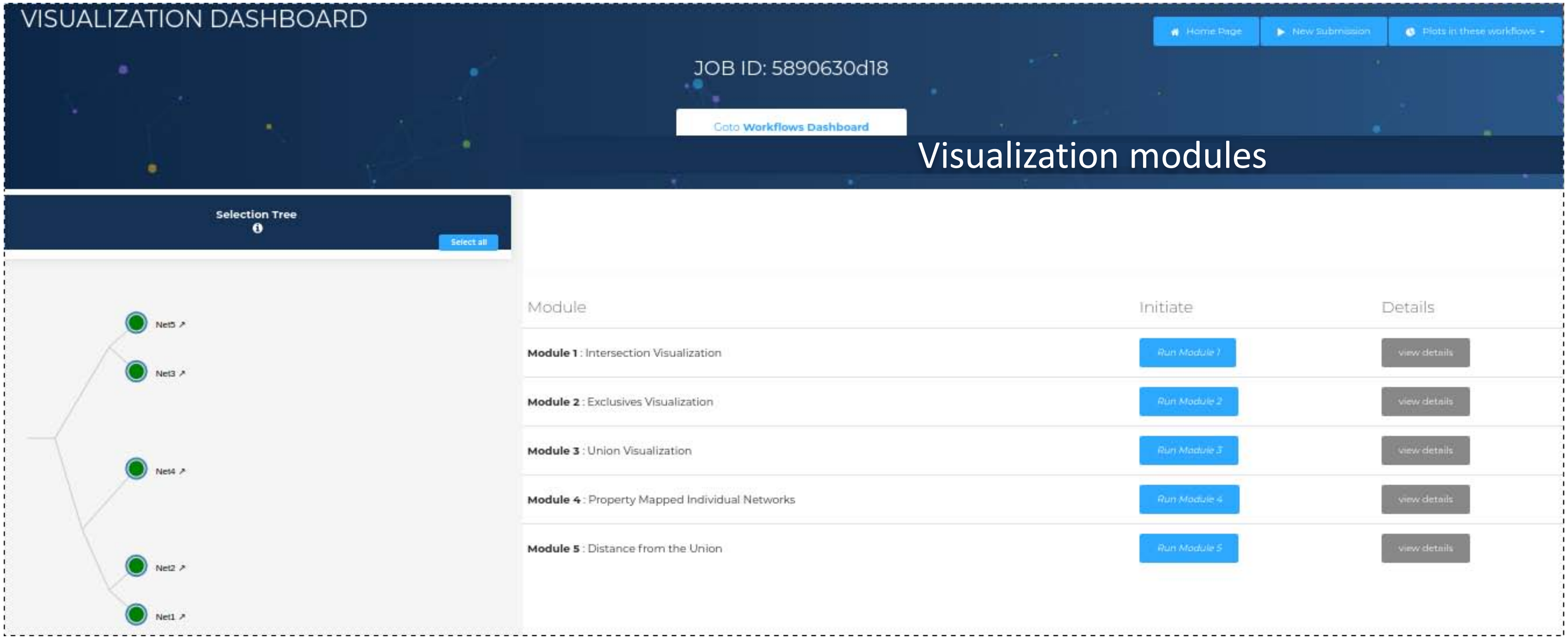


The visualization dashboard can be used to select two or more network from the tree and view a merged network of the intersection, union or exclusive

**Figure S17 :** The visualization dashboard

- **Nodes Pie** : Shows the presence of the node across the selected networks
- **Edge gradient** : Shows the presence of the edge across the selected networks

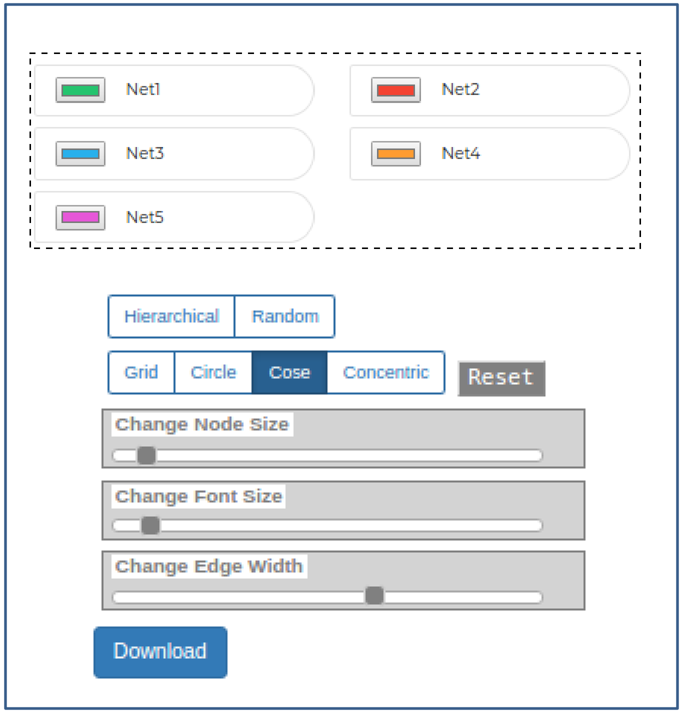


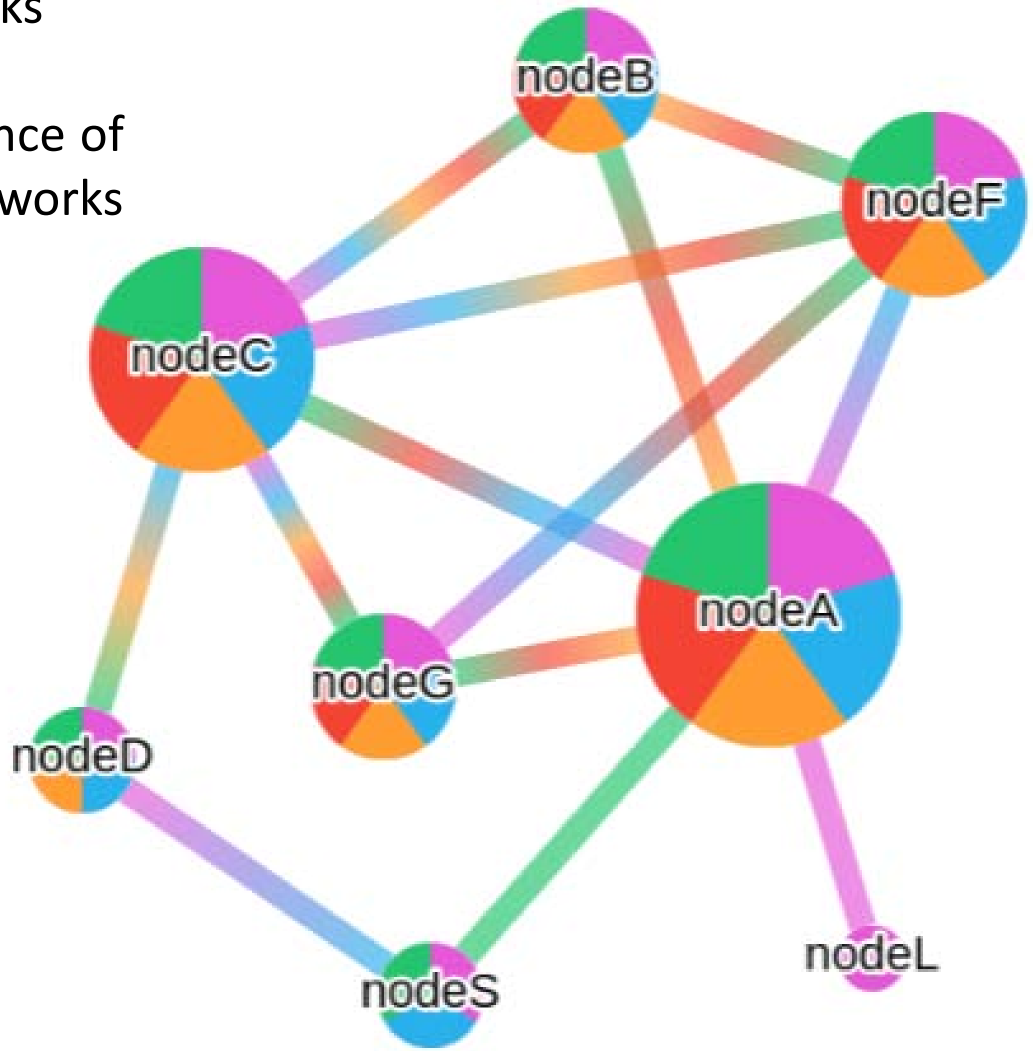


**Figure S18** : Visualizing union network with pie nodes

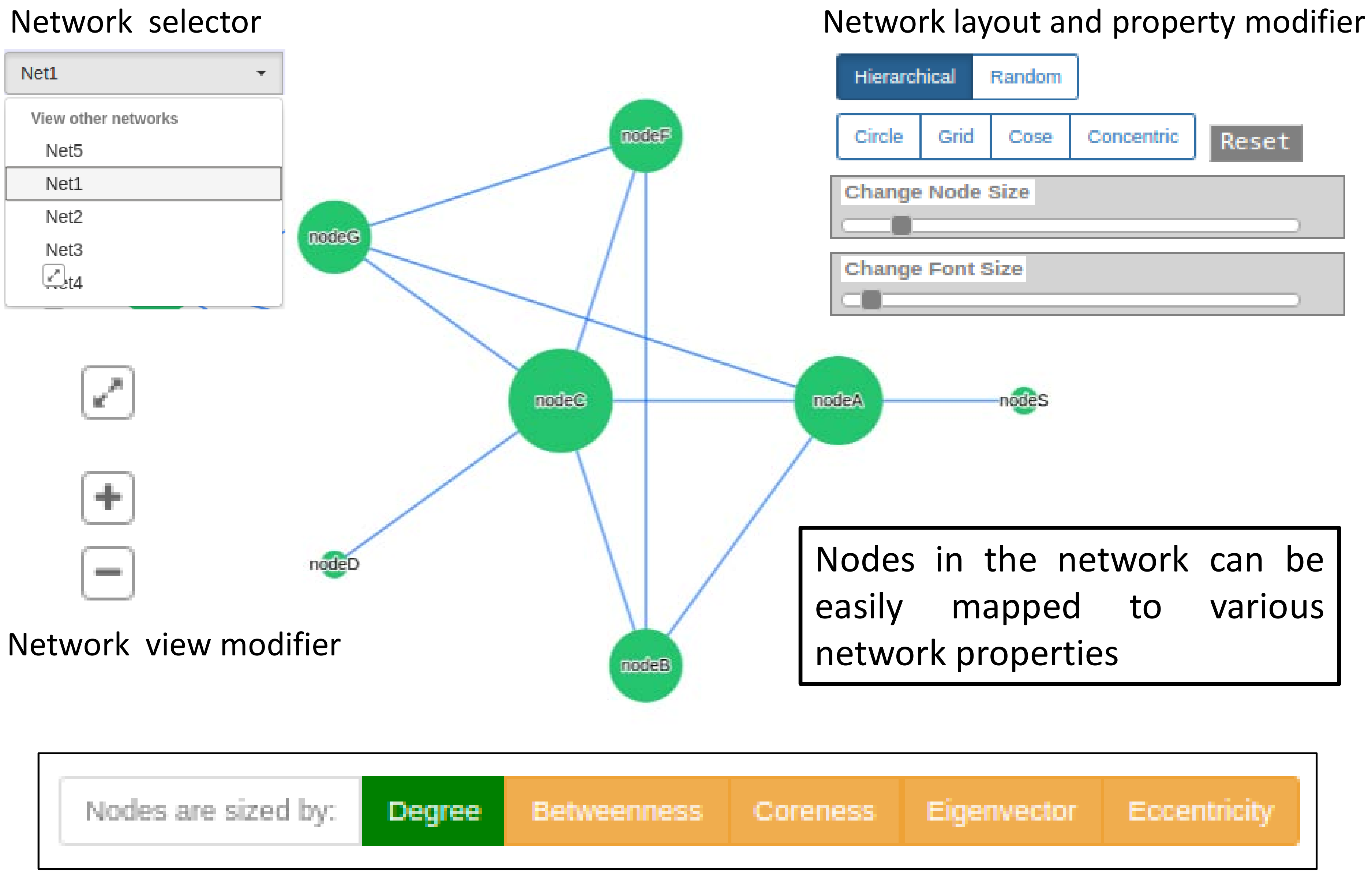


**Figure S19** : Network property and view modifier

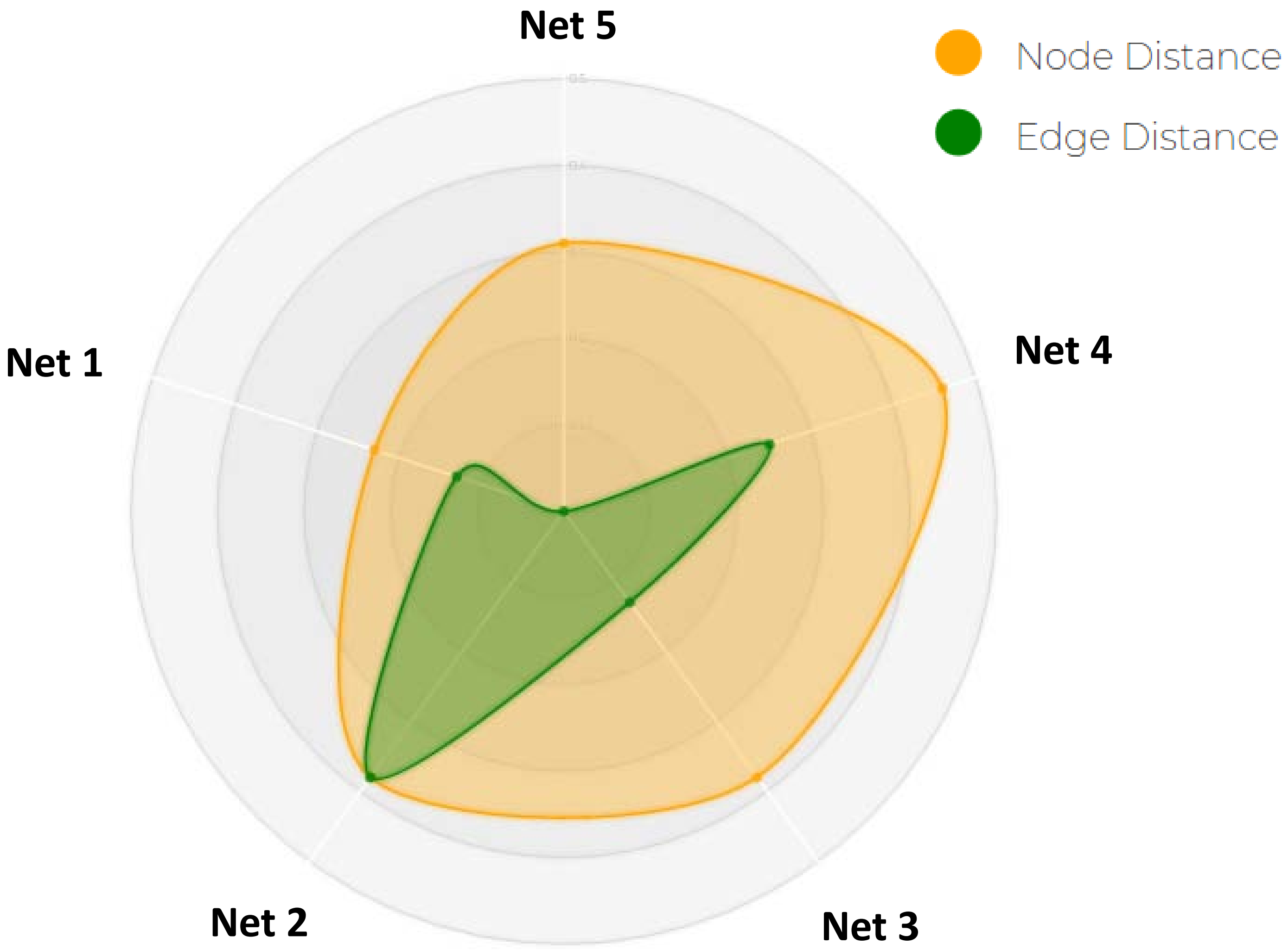


**Figure S20** : Distance from the global union network
