# Supplementary material for: NetConfer: a web application for comparative analysis of multiple biological networks
Source: BMC Biol. 2020 May 19;18:53. doi: 10.1186/s12915-020-00781-9 (PMC7236966; doi:10.1186/s12915-020-00781-9)
Supplement: Supplementary file 3 — Additional file 3: Fig. S21-S30. List of figures corresponding to ‘case study 1’ as described in the manuscript. [file 12915_2020_781_MOESM3_ESM.pdf]

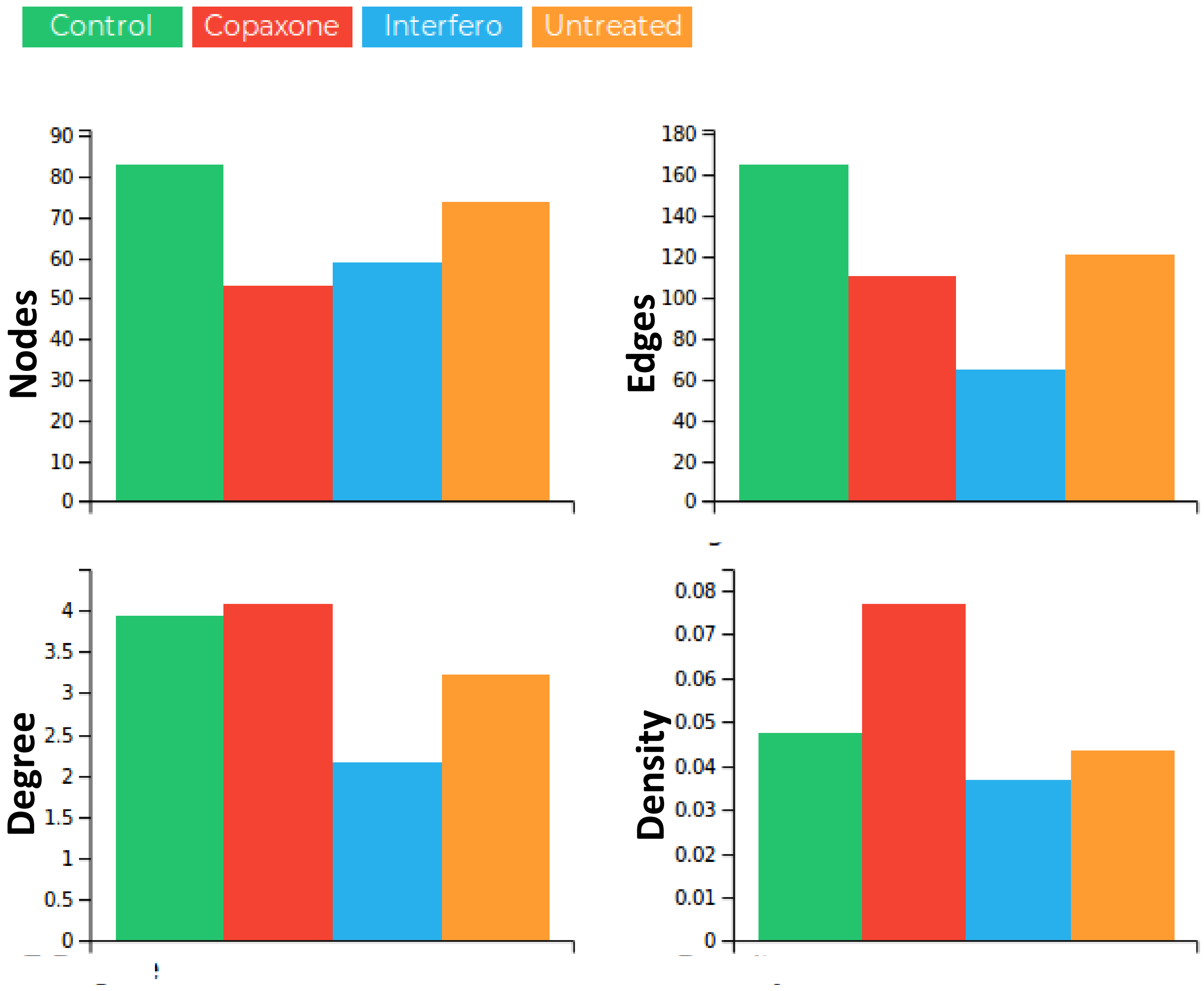

Figure S21 : Overview of the microbial association networks

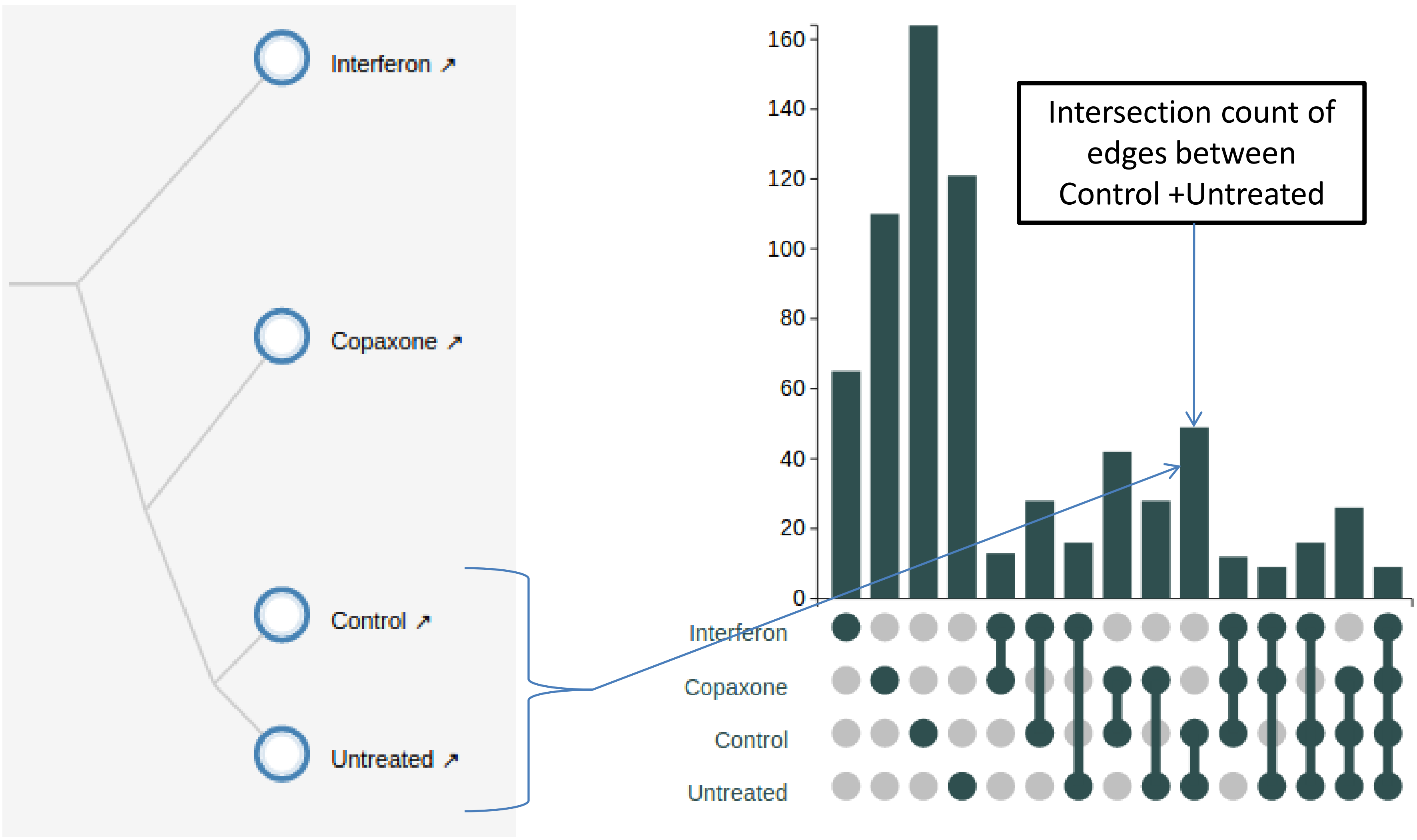

Figure S22 : Control and Untreated looks more similar based on Jaccard edge index. Every network had a good share of exclusive edges

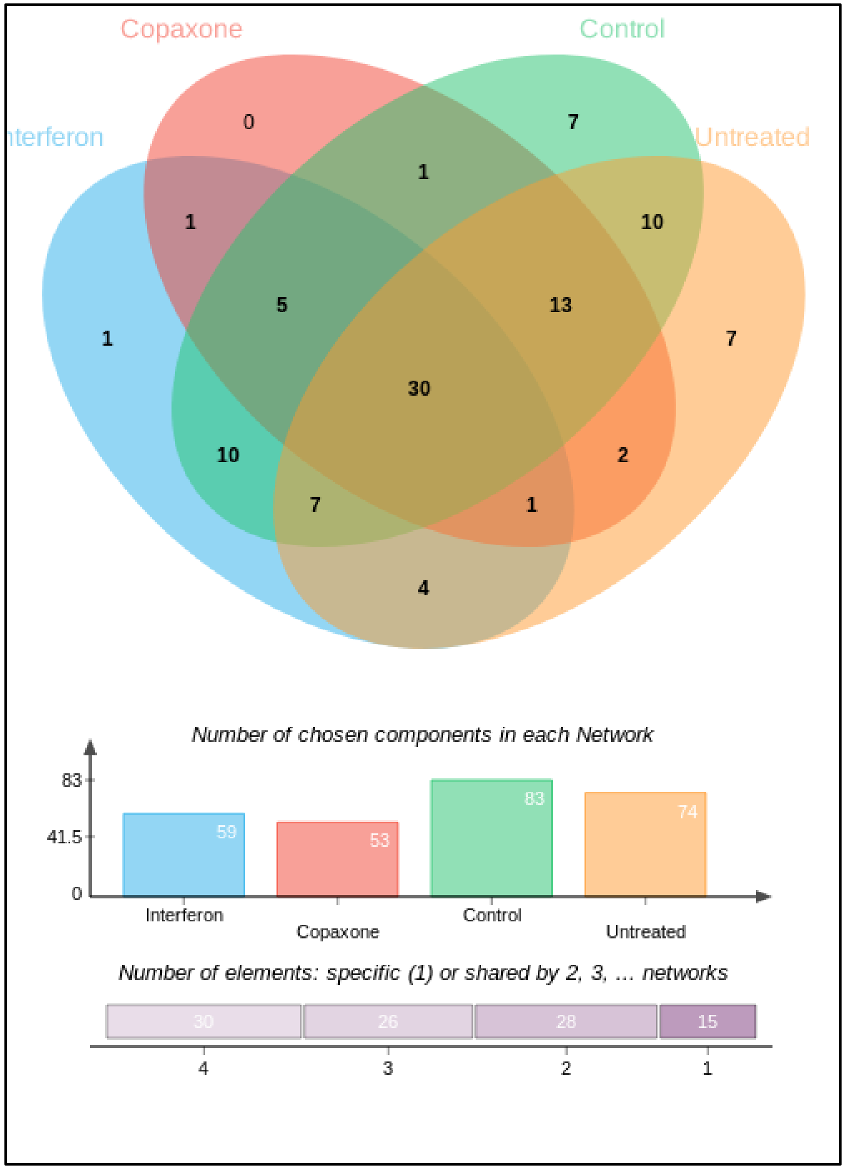

Nodes

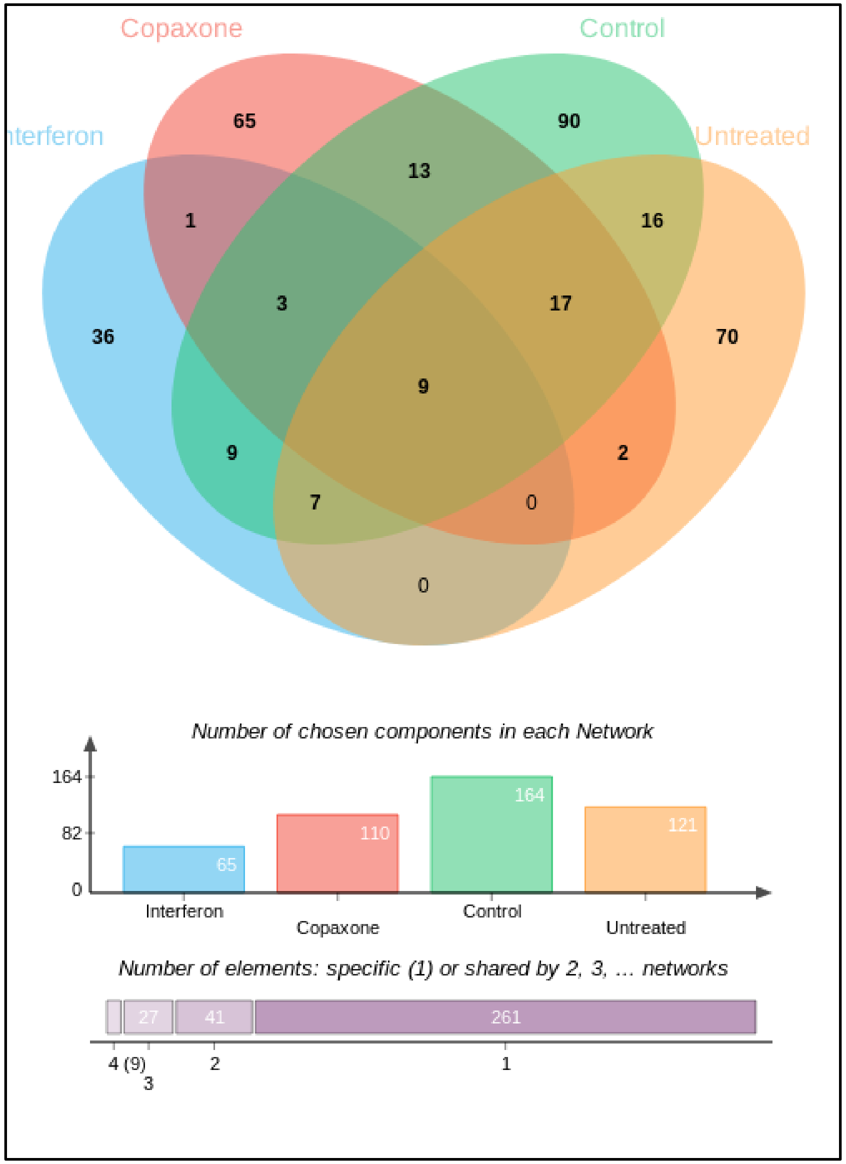

Edges

Figure S23 : Given a comparable set of nodes, many exclusive edges for each network hints towards a rewiring

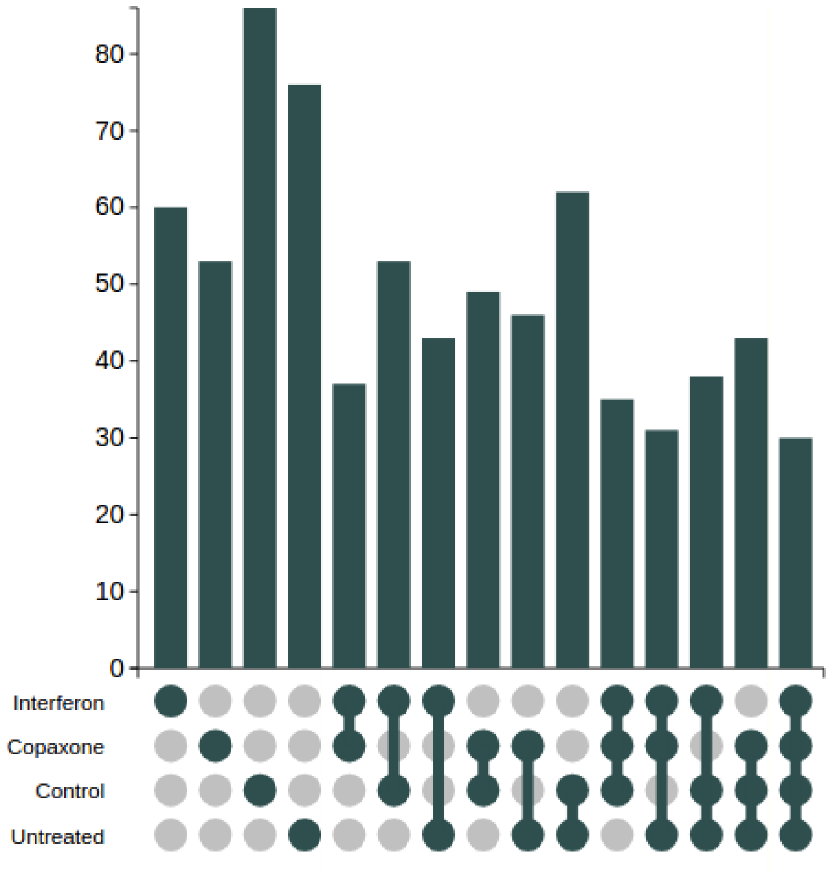

Node

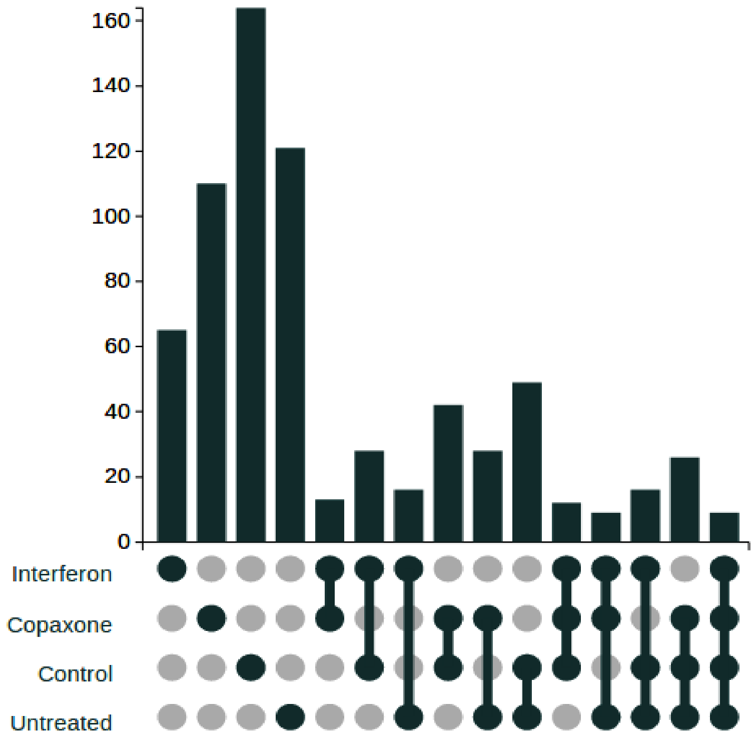

Edge

Figure S24 : A more clearer view of nodes being more similar than edges

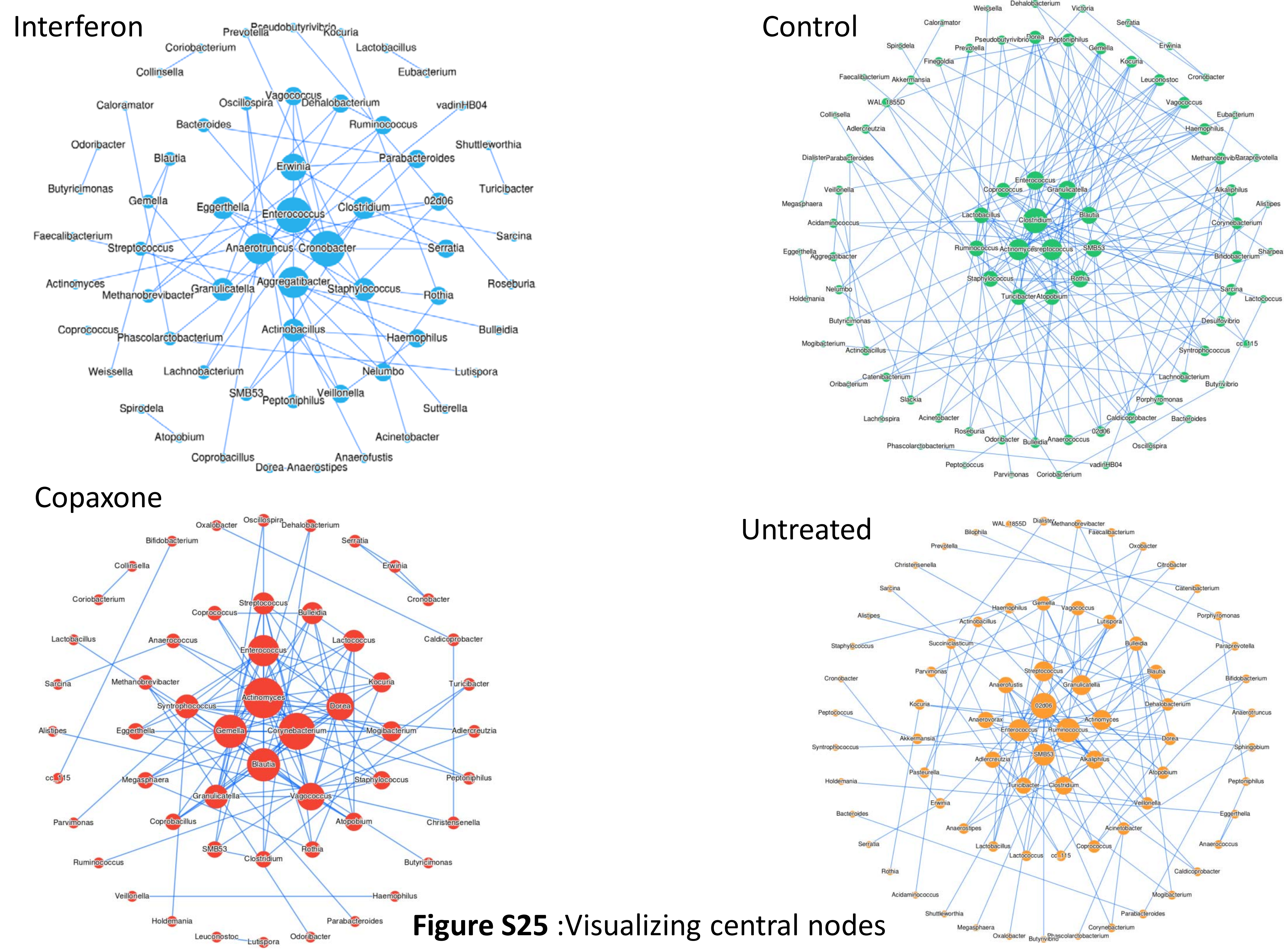

Figure S25 :Visualizing central nodes

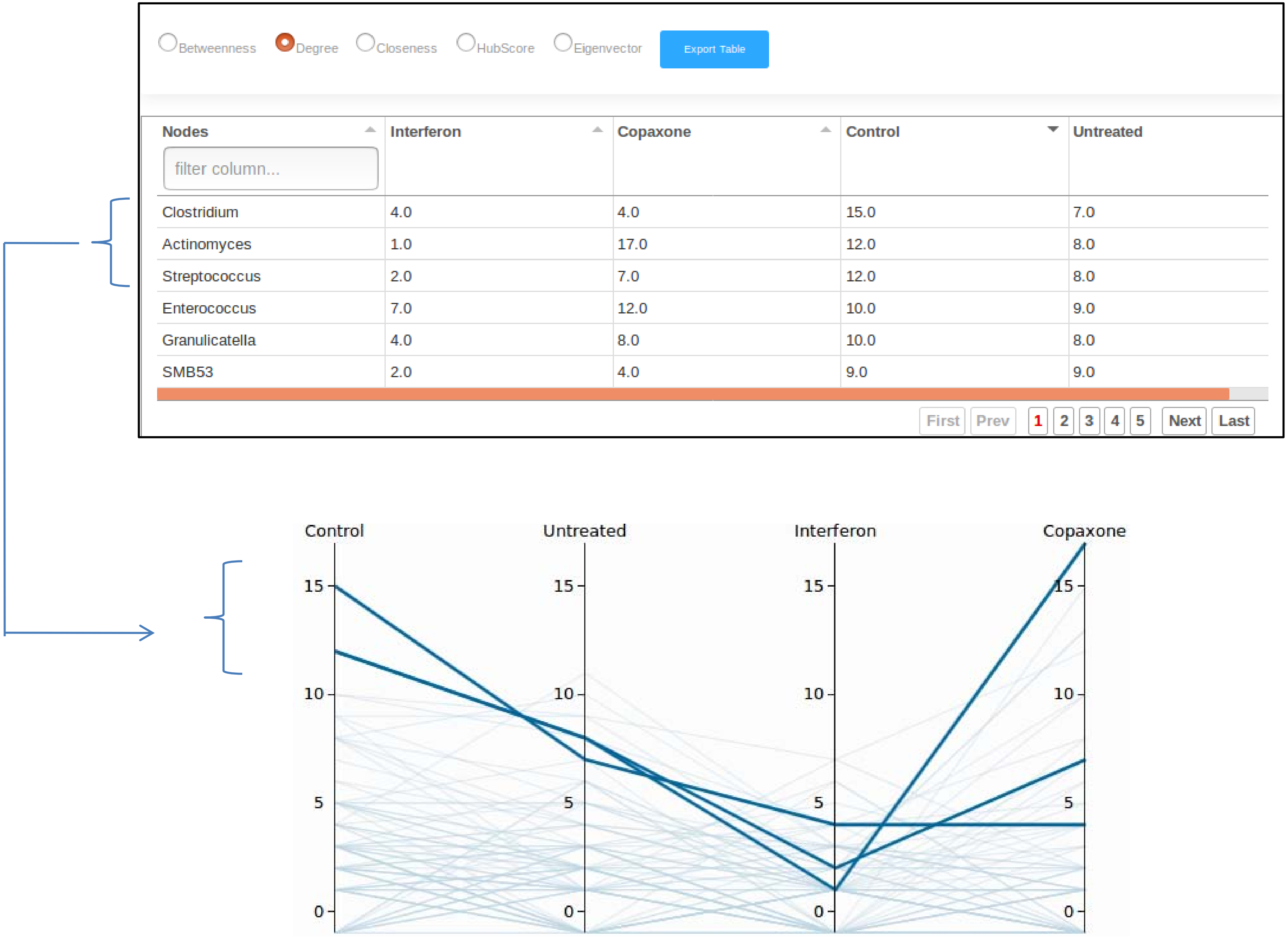

Nodes	Interferon	Copaxone	Control	Untreated
Clostridium	4.0	4.0	15.0	7.0
Actinomyces	1.0	17.0	12.0	8.0
Streptococcus	2.0	7.0	12.0	8.0
Enterococcus	7.0	12.0	10.0	9.0
Granulicatella	4.0	8.0	10.0	8.0
SMB53	2.0	4.0	9.0	9.0

Figure S26 : Visualizing changes in degree centrality across networks

Pathogen communications inferred using shortest paths

Network → Path ↓	Control	Copaxone	Interferon
Staphylococcus	1	1	2
Enterococcus			1
Streptococcus	0	0	0

Network → Path ↓	Control	Copaxone	Copaxone	Copaxone	Copaxone	Copaxone	Copaxone	Interferon	Untreated
Enterococcus	1	2	2	2	2	2	2	1	1
Actinomyces						1			
Corynebacterium					1				
Gemella		1							
Vagococcus							1		
Kocuria			1						
Granulicatella				1					
Streptococcus	0	0	0	0	0	0	0	0	0

Network → Path ↓	Control	Copaxone	Copaxone	Copaxone	Interferon
Enterococcus	1	2	2	2	1
Vagococcus				1	
Gemella		1			
Granulicatella			1		
Staphylococcus	0	0	0	0	0

Figure S27 : When inferred for pathogen communications (*Staphylococcus*, *Streptococcus* and *Enterococcus*), varying ways of shortest paths were seen

enterococcus staphylococcus

Export Clique Data

PREVIOUS 1 NEXT

Size	Network	CliqueID	Clique Membership
3	Interferon	Clique-9	Staphylococcus Eggerthella Enterococcus
6	Control	Clique-10	Streptococcus Rothia Granulicatella Enterococcus Staphylococcus Vagococcus

enterococcus streptococcus

Export Clique Data

PREVIOUS 1 NEXT

Size	Network	CliqueID	Clique Membership
3	Interferon	Clique-10	Granulicatella Enterococcus Streptococcus
3	Untreated	Clique-12	Enterococcus Lactobacillus Streptococcus
4	Control	Clique-14	Streptococcus Leuconostoc Actinomyces Enterococcus
4	Untreated	Clique-11	Enterococcus Atopobium Actinomyces Streptococcus
5	Untreated	Clique-8	Enterococcus Granulicatella Actinomyces Gemella Streptococcus
6	Control	Clique-9	Streptococcus Rothia Granulicatella Enterococcus Actinomyces Kocuria
6	Control	Clique-10	Streptococcus Rothia Granulicatella Enterococcus Staphylococcus Vagococcus

Figure S28: Clique analysis reveals the ‘partners in crime’

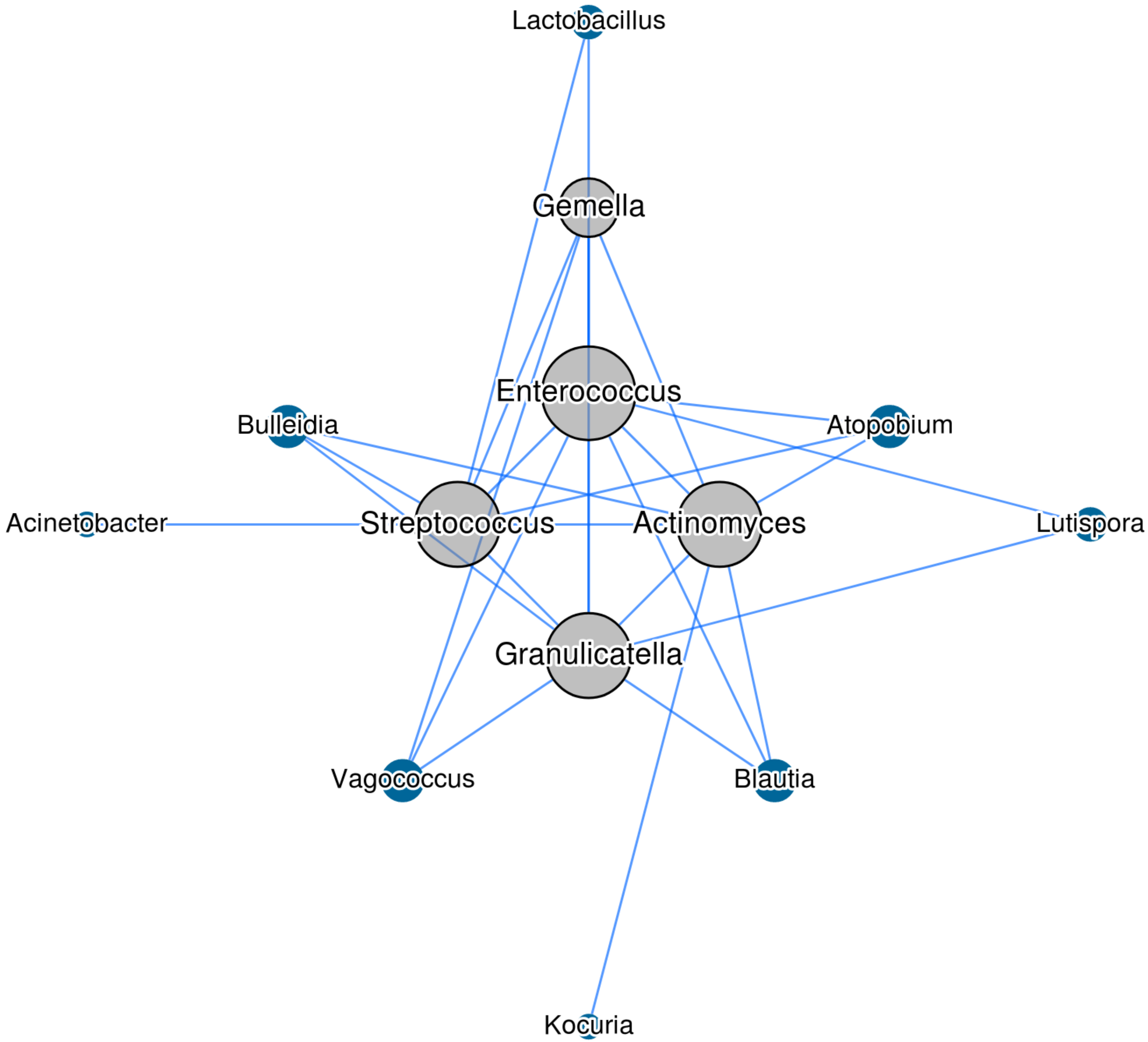

Figure S29: Visualizing a query clique with their first neighbors highlighted in blue. Node sizes mapped to their degree

Control -> Untreated

Untreated -> Interferon

Untreated -> Copaxone

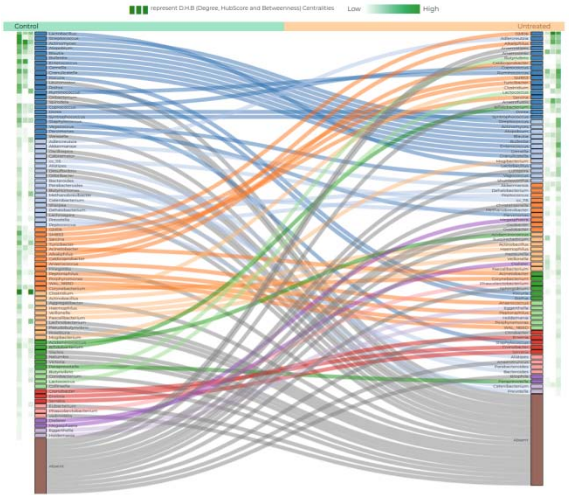

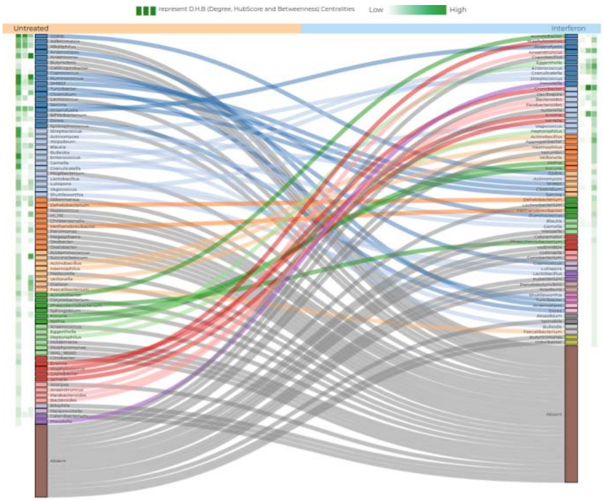

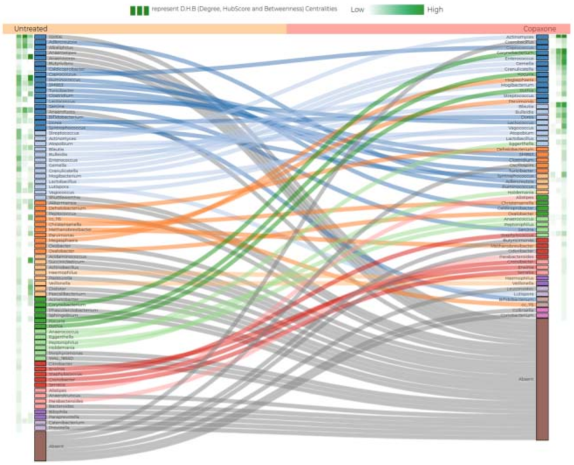

Little change

Maximum change

Moderate change

Weighted Jaccard Score = 3.8

Weighted Jaccard Score = 1.87

Weighted Jaccard Score = 3.03

Figure S30 : Varying levels of community shuffling evident in the networks. Lower 'Weighted Jaccard Score' indicates higher community shuffling
