# Supplementary material for: NetConfer: a web application for comparative analysis of multiple biological networks
Source: BMC Biol. 2020 May 19;18:53. doi: 10.1186/s12915-020-00781-9 (PMC7236966; doi:10.1186/s12915-020-00781-9)
Supplement: Supplementary file 4 — Additional file 4: Fig. S31-S37. List of figures corresponding to ‘case study 2’ as described in the manuscript. [file 12915_2020_781_MOESM4_ESM.pdf]

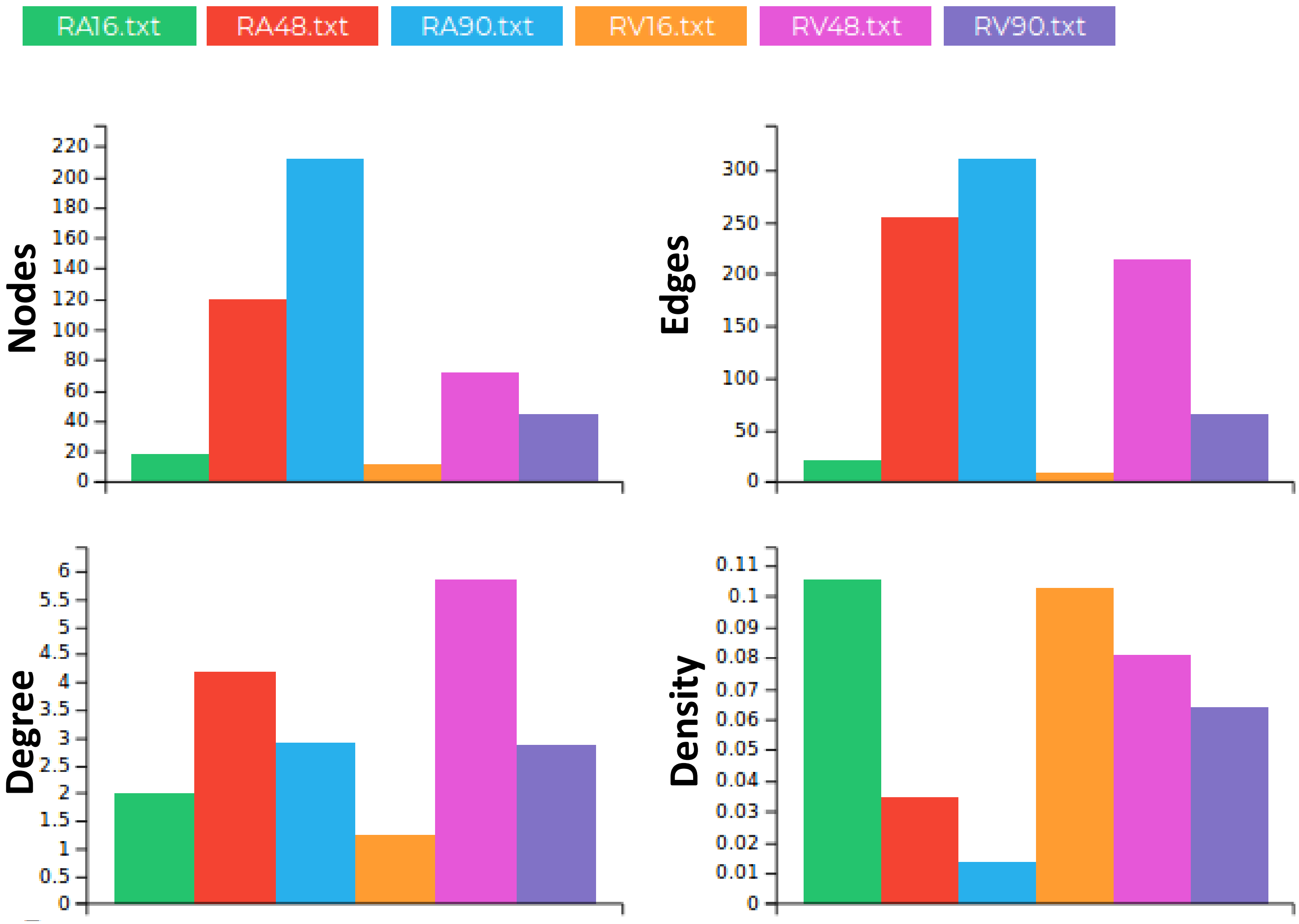

Figure S31: Global graph properties of four time series human gene perturbation (16hr, 48hr and 90hr) networks corresponding to avirulent (RA) and virulent (RV) strains

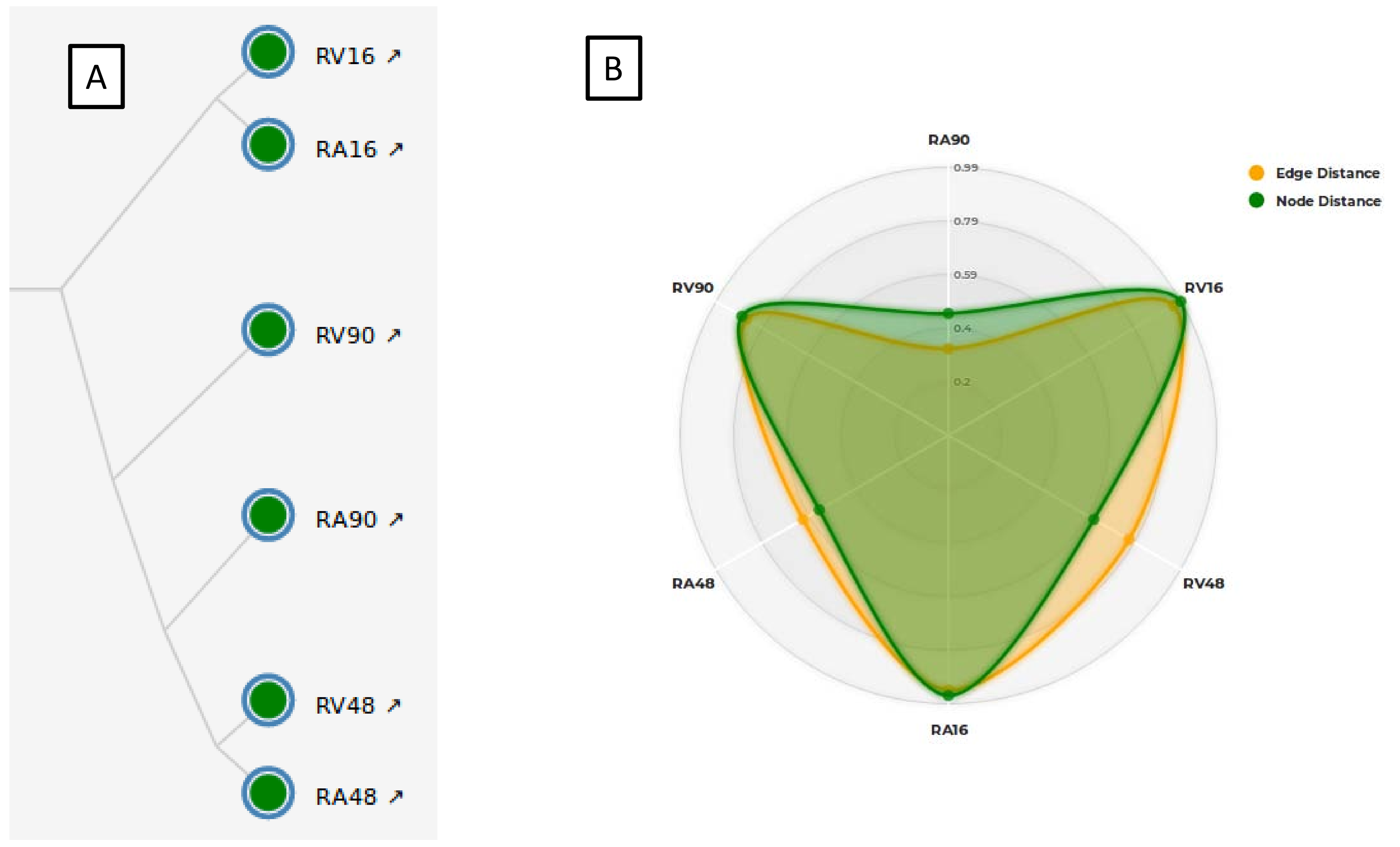

Figure S32: Comparison of the networks using (A) cluster network tree with Jaccard Edge distance and (B) radar plot of node and edge Jaccard distance from the union network

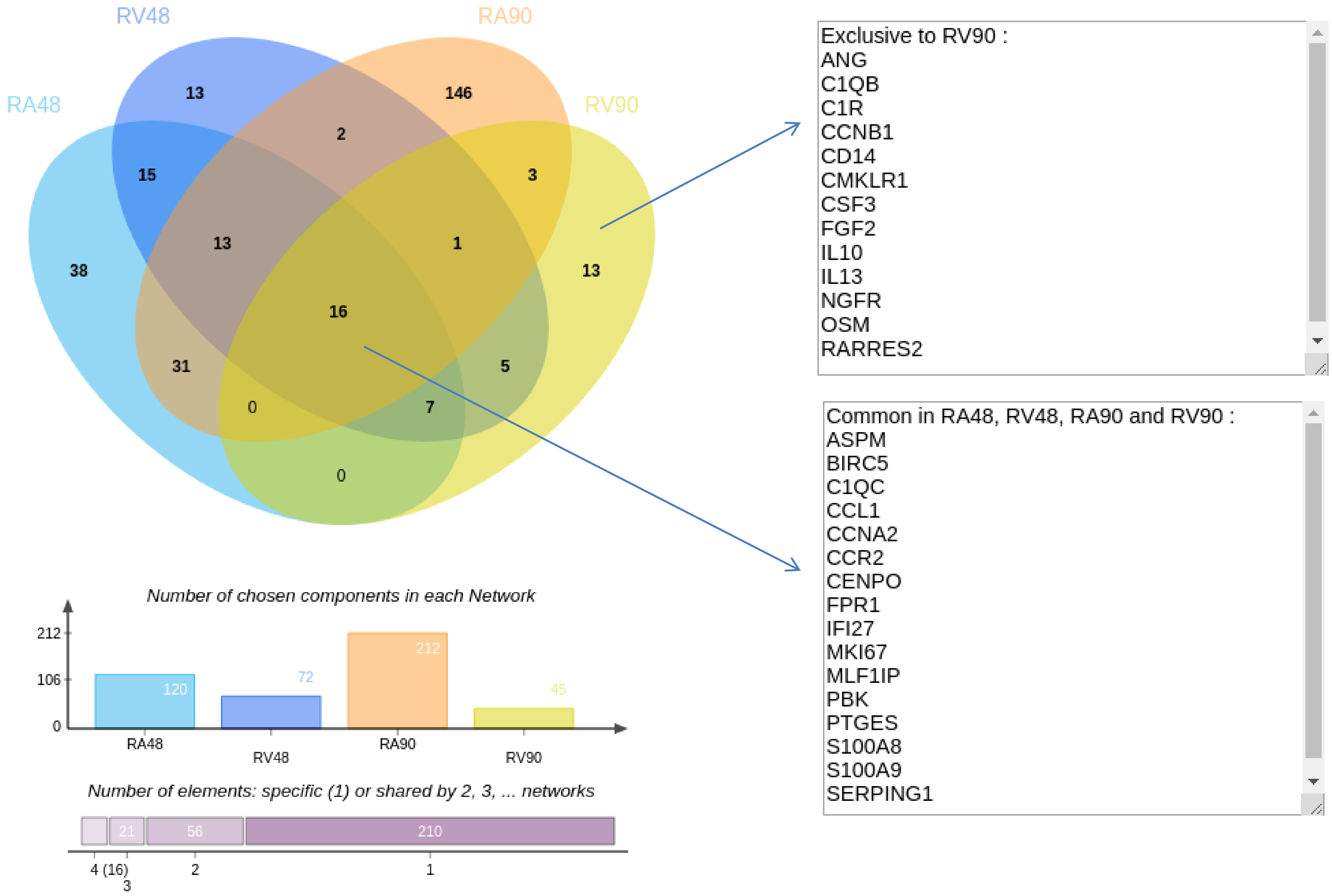

Figure S33: Visualizing similarities and differences between the post infection time point networks in H37Ra and H37Rv

A

Betweenness Degree Closeness HubScore Eigenvector Export Table

Sorted by Rv90 from High to Low values

Nodes	RV90	RA90	RV48	RA48
filter column...				
BIRC5	26.3	2584.531	201.091	178.442
CCNB1	23.533	-1.0	-1.0	-1.0
CCNA2	20.133	265.46	385.394	380.126
ASPM	14.0	0.0	1.283	3.417
CENPA	13.467	-1.0	46.943	42.096
NDC80	11.983	-1.0	23.412	72.162

(-1 values indicate node absence)

First Prev 1 2 3 4 5 Next Last

B

RV90 RA90 RV48 RA48 Export Table

Node	Degree	Closeness	Betwee...	EigenVe...	Network...	Clusteri...	PageRank	HubScore	Authority...
BIRC5	10.0	0.714	26.3	0.429	0.281	0.422	0.031	0.374	0.425
CCNB1	9.0	0.714	23.533	0.392	0.313	0.444	0.017	0.557	0.007
CCNA2	8.0	0.652	20.133	0.339	0.317	0.429	0.018	0.473	0.13
ASPM	3.0	0.5	14.0	0.118	0.425	0.333	0.025	0.0	0.227
CENPA	7.0	0.625	13.467	0.322	0.358	0.524	0.041	0.0	0.544
NDC80	7.0	0.625	11.983	0.319	0.354	0.524	0.013	0.416	0.123

First Prev 1 2 3 4 5 Next Last

Figure S34: (A) Visualizing nodes sorted with betweenness across various networks (B) visualizing variation of other network properties of the same nodes

Size	Network	CliqueID	Clique Membership
13	RV48	Clique-4	BIRC5 BUB1B NDC80 CDCA8 CENPM CENPE SPC25 CENPA CENPF ZWINT CENPN MLF1IP AURKB
13	RV48	Clique-5	BIRC5 BUB1B NDC80 CDCA8 CENPM CENPE SPC25 CENPA CENPF ZWINT CENPN MLF1IP KIF18A
13	RV48	Clique-6	BIRC5 BUB1B NDC80 CDCA8 CENPM CENPE SPC25 CENPA CENPF ZWINT CENPN NUF2 AURKB
6	RV48	Clique-0	BIRC5 BUB1B CCNA2 CENPA CENPE CDCA8
6	RV48	Clique-2	BIRC5 BUB1B CCNA2 PBK NCAPG RRM2
5	RV90	Clique-1	BIRC5 CCNB1 CCNA2 MKI67 PBK
5	RV90	Clique-2	BIRC5 CCNB1 CCNA2 MKI67 CDKN3
5	RV90	Clique-5	BIRC5 MLF1IP NDC80 KIF18A CENPA
5	RV48	Clique-1	BIRC5 BUB1B CCNA2 PBK CDCA8
5	RV48	Clique-7	BIRC5 BUB1B NDC80 CDCA8 PBK

Figure S35: Clique containing BIRC5 in the virulent strain infection networks

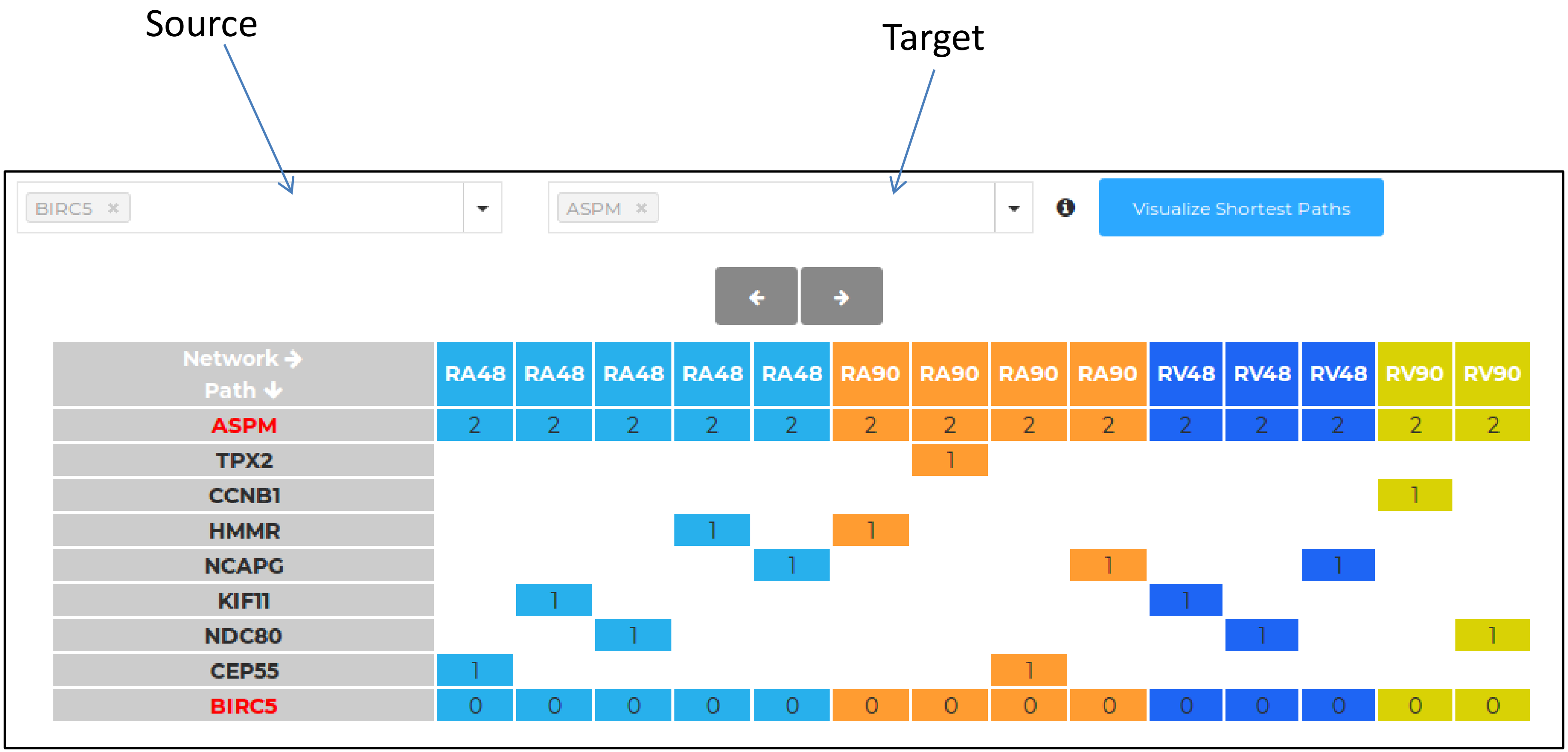

Network → Path ↓	RA48	RA48	RA48	RA48	RA48	RA90	RA90	RA90	RA90	RV48	RV48	RV48	RV90	RV90
ASPM	2	2	2	2	2	2	2	2	2	2	2	2	2	2
TPX2							1							
CCNB1													1	
HMMR				1		1								
NCAPG					1				1			1		
KIF11		1								1				
NDC80			1								1			1
CEP55	1							1						
BIRC5	0	0	0	0	0	0	0	0	0	0	0	0	0	0

Figure S36: Visualizing differential shortest paths for connected perturbation

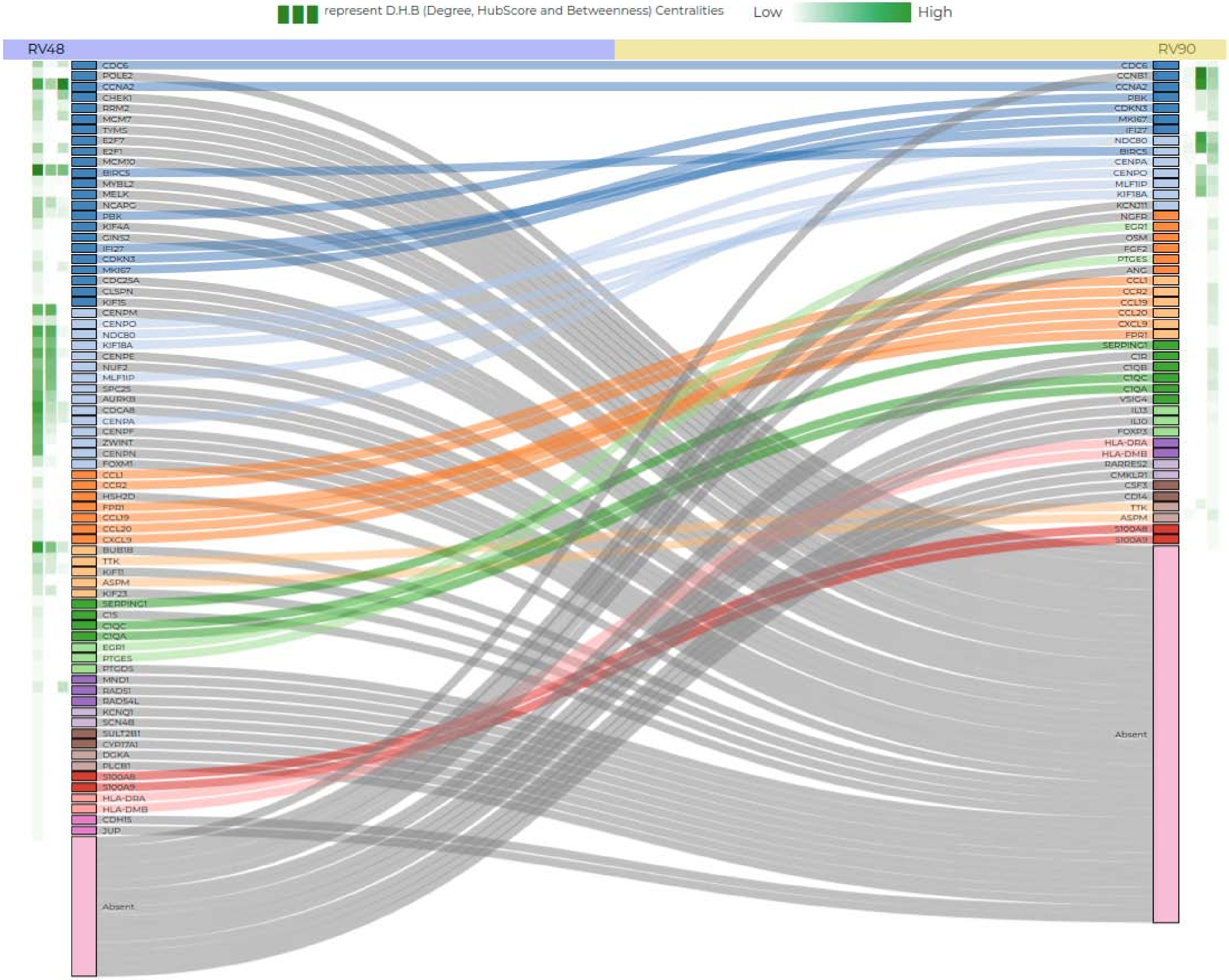

Figure S37: Visualizing changes in community structure between the 48th and 90th hour network of H37Rv infected human cells
