# Supplementary material for: NetConfer: a web application for comparative analysis of multiple biological networks
Source: BMC Biol. 2020 May 19;18:53. doi: 10.1186/s12915-020-00781-9 (PMC7236966; doi:10.1186/s12915-020-00781-9)
Supplement: Supplementary file 5 — Additional file 5: Table 2. Time evaluation of computationally intensive modules in NetConfer. [file 12915_2020_781_MOESM5_ESM.pdf]

Uploading and global property calculation		Edges (n * fold) - Time in seconds			
		n*2	n*3	n*4	n*5
Nodes (n)	500	0.759	1.48	1.469	1.54
	1000	2.04	1.93	1.89	2.26
	1500	2.502	2.52	2.65	2.7
	2000	2.96	3.26	3.46	3.61
	3000	4.87	5.49	5.87	5.68
	4000	7.62	8.24	8.86	9.52
	5000	11.42	11.8	12.52	13.59

Community finding		Edges (n * fold) - Time in seconds			
		n*2	n*3	n*4	n*5
Nodes (n)	500	3.47	3.72	3.55	3.98
	1000	4.72	5.09	5.31	5.614
	1500	6.36	6.55	7.47	7.99
	2000	7.69	9.455	9.68	11.93
	3000	12.2	14.39	17.31	22.04
	4000	16.89	23.72	29.86	35.1
	5000	23.14	32.88	41.59	51.75

Local graph properties calculation		Edges (n * fold) - Time in seconds			
		n*2	n*3	n*4	n*5
Nodes (n)	500	3.58	3.77	3.98	4.32
	1000	6.03	6.82	7.34	9.21
	1500	10.27	12.69	14.59	16.62
	2000	13.17	18.06	20.56	23.86
	3000	29.13	38.04	45.58	53.96
	4000	46.55	56.57	70.92	83.175
	5000	74.21	96.67	115.82	134.81

Clique finding		Edges (n * fold) - Time in seconds			
		n*2	n*3	n*4	n*5
Nodes (n)	500	3.553	4.1	6.45	8.6
	1000	4.24	6.13	6.94	9.35
	1500	6.32	7.85	9.08	13.02
	2000	7.6	9.232	12.87	16.62
	3000	12.59	17.5	20.07	26.54
	4000	18.18	23.73	31.86	38.14
	5000	26.17	33.83	42.411	55.19

Note : The time reported in the above table might vary depending on server load and future upgrades.

Table 2: Time evaluation of computationally intensive modules in NetConfer
